# Integrated Taxonomy Revealed Genetic Differences in Morphologically Similar and Non-Sympatric *Scoliodon macrorhynchos* and *S. laticaudus*

**DOI:** 10.3390/ani12060681

**Published:** 2022-03-08

**Authors:** Kean Chong Lim, William T. White, Amy Y. H. Then, Gavin J. P. Naylor, Sirachai Arunrugstichai, Kar-Hoe Loh

**Affiliations:** 1 Institute of Ocean and Earth Sciences, Universiti Malaya, Kuala Lumpur 50603, Malaysia; keanchonglim@gmail.com; 2 Institute of Advanced Studies, Universiti Malaya, Kuala Lumpur 50603, Malaysia; 3 CSIRO National Research Collections Australia, Australia National Fish Collection, Hobart, TAS 7001, Australia; william.white@csiro.au; 4 Institute of Biological Sciences, Universiti Malaya, Kuala Lumpur 50603, Malaysia; 5 Florida Museum of Natural History, Dickinson Hall, Gainesville, FL 32601, USA; gjpnaylor@gmail.com; 6 Aow Thai Marine Ecology Centre, Bangkok 10100, Thailand; carcharodon.shinalodon@gmail.com

**Keywords:** spadenose sharks, integrated taxonomy, synonymy, Indo-West Pacific, morphometrics, genetics, distribution range

## Abstract

**Simple Summary:**

In this study, the species identities of similar-looking coastal spadenose sharks from different areas were clarified by adding new molecular markers and more individual body measurements, including animals from the Malaysian Peninsula that had not been examined previously. Collective evidence showed that there are two genetically distinct species that do not overlap in their spatial occurrence. The Malacca Strait acts as a boundary delineating the distribution range of the Pacific spadenose shark *Scoliodon macrorhynchos* to the east and, of the Northern Indian Ocean, *S. laticaudus* to the west. In addition, the need to determine the species status of *Scoliodon* animals from Indonesian waters was identified. The present study reinforced the need to rely on comprehensive genetic information in addition to external characteristics to assess the species identities and distribution range for small sharks and rays that have apparent contiguous coastal distribution with limited dispersal abilities.

**Abstract:**

Previous examination of the mitochondrial *NADH2* gene and morphological characteristics led to the resurrection of *Scoliodon macrorhynchos* as a second valid species in the genus, in addition to *S. laticaudus*. This study applied an integrated taxonomic approach to revisit the classification of the genus *Scoliodon* based on new materials from the Malaysian Peninsula, Malaysian Borneo and Eastern Bay of Bengal. Mitochondrial DNA data suggested the possibility of three species of *Scoliodon* in the Indo-West Pacific, while the nuclear DNA data showed partially concordant results with a monophyletic clade of *S. macrorhynchos* and paraphyletic clades of *S. laticaudus* and *S.* cf. *laticaudus* from the Malacca Strait. Morphological, meristic and dental characteristics overlapped between the three putative species. Collective molecular and morphological evidence suggested that the differences that exist among the non-sympatric species of *Scoliodon* are consistent with isolation by distance, and *Scoliodon macrorhynchos* remains as a valid species, while *S.* cf. *laticaudus* is assigned as *S. laticaudus*. The Malacca Strait acts as a spatial delineator in separating the Pacific *S. macrorhynchos* (including South China Sea) from the Northern Indian Ocean *S. laticaudus*. Future taxonomic work should focus on clarifying the taxonomic status of *Scoliodon* from the Indonesian waters.

## 1. Introduction

The genus *Scoliodon* was proposed by Müller and Henle [1] for *S. laticaudus* Müller and Henle [2]. Within the family Carcharhinidae, this genus is distinguished from other genera by its clasper and cranial morphology and very shallowly concave post-ventral caudal margin [3]. The genus *Scoliodon* is morphologically similar to hammerhead sharks (family Sphyrnidae) in a number of proportional body measurements but is placed in Carcharhinidae, as it does not have the laterally expanded head that is characteristic of hammerheads [4]. The genus sits within the subfamily Scoliodontinae and differs from the other genera within the subfamily, i.e., *Rhizoprionodon* and *Loxodon*, by having a greatly depressed, trowel-shaped head, broader and more triangular pectoral fins and a more posteriorly located first dorsal fin (free rear tip about over mid-bases of the caudal fin) [5].

*Scoliodon* has long been considered to be a monotypic genus until White et al. [5] resurrected *S. macrorhynchos* [6] as a second species within the genus. *Scoliodon laticaudus* is common along the insular shelf extending from the Northern Indian Ocean to Northeastern Africa [7]. *Scoliodon macrorhynchos* is known from Southeast Asia from Taiwan and China to Indonesia and Sarawak, Malaysia [5]. A possible third species was also reported from the Bay of Bengal by White et al. [5] and Naylor et al. [8] based on *NADH2* sequence data. These authors suggested that *Carcharhias* (*Physodon*) *muelleri* Müller and Henle [2], described from Bengal may be an available name for this species, but in the absence of specimens, this species was not formally resurrected.

The spadenose shark is one of the smallest carcharhinid species, attaining a maximum total length of 74 cm [9], occurring in shallow muddy and sandy bottom habitats [10]. Nearshore elasmobranchs generally have limited dispersal capabilities [4]. For instance, the bambooshark *Chiloscyllium punctatum* [11] and the stingray *Neotrygon* species [12], both of which are small, show regional population subdivisions with limited genetic mixing throughout the Indo-West Pacific. When geographic barriers and the lack of suitable contiguous habitats are combined with a proclivity not to disperse, allopatric speciation becomes more likely. These factors influenced the redescription of *S. macrorhynchos* from the Eastern South China Sea and the suggestion that *S. muelleri* from the Bay of Bengal might also be a distinct species [5].

White et al. [5] found that *S. macrorhynchos* and *S. laticaudus* showed high intraspecific variations from morphometric data (as high as 5.2% in some head and snout measurements) but low interspecific variations; only a limited number of morphometric measurements differed between the two species, with partly overlapping ranges. For the molecular analysis, the interspecific genetic distance of the *NADH dehydrogenase subunit 2* (*NADH2*) gene between *S. macrorhynchos* and *S. laticaudus* was about 3%. This degree of divergence falls at the borderline of “intra” versus “inter”-specific genetic variations in sharks and rays. *Mobula kuhlii* and *M. eregoodoo* were viewed as one species based on their close genetic distance (interspecific distance < 1.5%) but viewed as distinct species based on morphological data [13]. *Hypanus berthalutzae* was viewed as a distinct species from other closely related *Hypanus* species based on genetics (interspecific distance 0.82–3.11%), morphology, and ecological niche modeling data [14]. These examples highlight the challenge of distinguishing similar-looking but potentially distinct species, such as those in the genus *Scoliodon*.

Reliance on mitochondrial DNA (mtDNA) alone in elucidating phylogenetic relationships among closely related species has been called into question. Reviews by Galtier et al. [15] and Balloux [16] presented some of the limitations associated with reliance on mitochondrial data. The concerns raised arose from limited cases of non-maternally transmitted mtDNA that may call into question the assumption of reduced within-individual diversity [17,18,19], non-neutral evolution through selection [20,21,22], and the nonconstant mutation rate in mtDNA [23,24,25]. While these concerns may not necessarily be applicable in the representation of within-species history for *Scoliodon*, the genetic basis for delineating *S. macrorhynchos* as a separate species from *S. laticaudus* [5] merits a critical review using more representative sampling with additional markers.

In this study, both nuclear and mtDNA markers were used in addition to morphological data sample specimens across known geographical range of *Scoliodon* to clarify the phylogenetic relationships for the group. We included specimens of *Scoliodon* from the Malacca Strait, the west coast of Peninsula Malaysia, that had not been previously examined. The fine-scale contemporary distribution range of the *Scoliodon* genus, especially in the Indo-Malaya region, and knowledge gaps were discussed.

## 2. Materials and Methods

### 2.1. Sample Collection

Specimens of *Scoliodon* were acquired at fish landing sites located in the Malacca Strait on the west coast of Peninsular Malaysia, i.e., Hutan Melintang (3°52′13.6″ N 100°55′39.3″ E), Sungai Besar (3°40′15.2″ N 100°58′52.3″ E), and Pasir Penambang (3°21′03.9″ N 101°15′07.0″ E), henceforth labeled as *S.* cf. *laticaudus* and *S. macrorhynchos* from two landing sites in Sarawak in Malaysian Borneo, i.e., Kuching (1°34′04.7″ N, 110°22′45.8″ E) and Mukah (2°53′50.6″ N, 112°05′45.6″ E). Tissue samples were taken from a random subset of specimens (10 each from Malacca Strait and from Sarawak) and stored in 95% alcohol prior to molecular analyses, while the whole specimens were fixed using 10% formalin and store in 70% alcohol. A subset of specimens, 21 from Malacca Strait and 13 from Sarawak, was preserved whole and retained for subsequent morphological analysis by one of us (KCL). Eleven whole specimens of *S.* cf. *laticaudus* were also collected from the Ranong fishing port in Thailand, Eastern Bay of Bengal, during recent surveys of that landing site [26].

### 2.2. Molecular Analyses

Two mitochondrial DNA (*cytochrome oxidase subunit 1* (*COI*) and *NADH dehydrogenase subunit 2* (*NADH2*) regions) were used in molecular species identification and seven nuclear genes following Aschliman et al. [27] DNA (*actin-like protein* (*ACT*), *kelch repeat and BTB domain-containing protein 2* (*KBTBD2*), *prospero homeobox protein 1* (*PROX1*), *recombination activating gene 1* (*RAG1*), *recombination activating gene 2* (*RAG2*), *sec1 family domain-containing protein 2* (*SCFD2*), and *transducer of ERBB2.1* (*TOB1*) region) were used to verify the taxonomic assignment using mitochondrial DNA. DNA was extracted using 10% Chelex resin incubated for two minutes at 60 °C, followed by 25 min at 103 °C (modified from Hyde et al. [28]). Extracted DNA was subjected to Polymerase Chain Reaction (PCR) to amplify all targeted DNA markers. PCR were carried out either using iTaq^TM^ Plus DNA Polymerase (iNtRON Biotechnology, INC., Seongnam-si, Korea) or MyTaq^TM^ Red Mix (Bioline, London, United Kingdom) in 20 µL of reaction mix containing 2 µL of 10x PCR buffer; 0.5 µL of dNTP mixture (2.5 mM each); 1 µL of 10-pmol primer (both primers); 1.25 unit of Taq DNA polymerase; 1 µL of 50-pg–1.0-µg DNA templates; and top up with molecular-grade water or 10 µL of MyTaq^TM^ Red Mix premix (mixture of 10x PCR buffer, dNTPs, and Taq polymerase); 1 µL of 10-pmol primer (both primers); 1 µL of 50-pg–1.0-µg DNA templates; and top up with molecular-grade water, respectively. The PCR cycles for mitochondrial DNA comprised of 2-min initial denaturation at 94 °C, followed by 30 cycles of 20 s at 94 °C, 20 s at 44 °C (*COI*) or 52 °C (*NADH2*), and 1 min at 72 °C and, subsequently, a final extension of 5 min at 72 °C. The PCR cycles for nuclear DNA comprised 3-min initial denaturation at 95 °C, followed by 35 cycles of 15 s at 95 °C, 15 s at 52–60 °C, and 1 min at 72 °C and, subsequently, a final extension of 5 min at 72 °C. Touchdown PCR with annealing temperature that decreased 0.3 °C/cycle from 68 °C to 58 °C was performed on *PROX1* due to the amplification of nonspecific DNA at all tested temperatures between 45 and 60 °C. The primer sets used for all the targeted regions are listed in Table 1. All PCR products were examined using 1% agarose in TAE buffer prior to the Sanger sequencing service at Apical Scientific Sdn Bhd (Selangor, Malaysia).

### 2.3. Phylogenetic Analysis

Sequences were reviewed manually using BioEdit [31], aligned using ClustalX [32], and finally, trimmed using BioEdit [31]. They were all submitted to the NCBI GenBank database, with the accession numbers provided in Supplementary Table S1. The following analyses applied to individual marker, as well as grouped markers by mitochondrial DNA and nuclear DNA. The aligned sequences were subjected to the best model search based on Akaike Information Criterion (AIC) and Bayesian Information Criterion (BIC) for Maximum Likelihood (ML) and Bayesian Inference (BI) analyses, respectively, using Kakusan v 3 [33], as shown in Supplementary Table S2. The generated files were subsequently used for phylogenetic tree construction using Treefinder for ML [34] and MrBayes for BI [35]. The ML analyses were performed with 1000 bootstrap replicates. The Bayesian analyses were initiated with a random starting tree and two parallel runs, each of which consisted of running four chains of Markov chain Monte Carlo (MCMC) iterations for 2,000,000 generations (sampled every 100th generation for each chain). The convergence and burn-in from “sump” commands in MrBayes were used to evaluate likelihood values for post-analysis trees and parameters. Five thousand trees generated were discarded as burn-in (where the likelihood values were stabilized prior before the burn-in), and the remaining trees after burn-in were used to calculate the posterior probabilities using the “sumt” command.

The finalized ML and BI phylogenetic trees were processed via Figtree v 1.3.1 (http://tree.bio.ed.ac.uk/software/figtree/, accessed on 1 October 2014). For mitochondrial DNA, sequences of closely related species *Loxodon macrorhinus* and *Lamiopsis tephrodes* were used as outgroups. Sequences of *Sphyrna lewini* and *Rhizoprionodon acutus*, on the other hand, were used as the outgroup for nuclear DNA, as the sequences for *Loxodon* and *Lamiopsis* were not available. As such, the sequence of *S. lewini* was added to mitochondrial DNA analyses to facilitate the comparison between mitochondrial and nuclear DNA. Some other sequences available in the National Center for Biotechnology Information (NCBI) GenBank and Barcode of Life Data (BOLD) systems were also used in the tree construction for comparison (Supplementary Table S3). Uncorrected p-distance was calculated using PAUP* 40b10 software [36] to evaluate the genetic divergence among the sampled *Scoliodon* species by sampling areas.

We tested species delimitation using a multispecies coalescent analysis implemented in ASTRAL 5.7.7 [37,38] and BPP 4.3 [39,40,41]. In the ASTRAL analysis, two hundred gene trees were searched under ML + rapid bootstrap for each of the genes using raxmlGUI 1.5 beta [42]. All generated gene trees were combined manually as input into ASTRAL to generate a ASTRAL species tree and normalized quartet score. The normalized quartet score refers to the proportion of gene trees that matched with the species tree; a higher score indicates greater agreement between gene trees and species tree. In the BPP analysis, we performed an unguided species delimitation analysis (A11) to test if the *Scoliodon* species can be assigned as a single species. We set multiple theta (population size) and tau (divergence time) combinations using the inverse gamma prior to IG (2, X), with X being 0.1, 0.01, and 0.001. Each analysis was performed twice to confirm the stability of the results.

### 2.4. Morphological and Meristic Data

Measurement terminology followed Compagno [3,4,43], who assigned names and abbreviations to measurements often indicated by descriptive phrases (example: snout to upper caudal origin = precaudal length = PRC). Dentitional terms generally followed Compagno [3,43,44]. Vertebral terminology, method of counting, and vertebral ratios followed Springer and Garrick [45] and Compagno [3,43,44].

**Figure 1:**
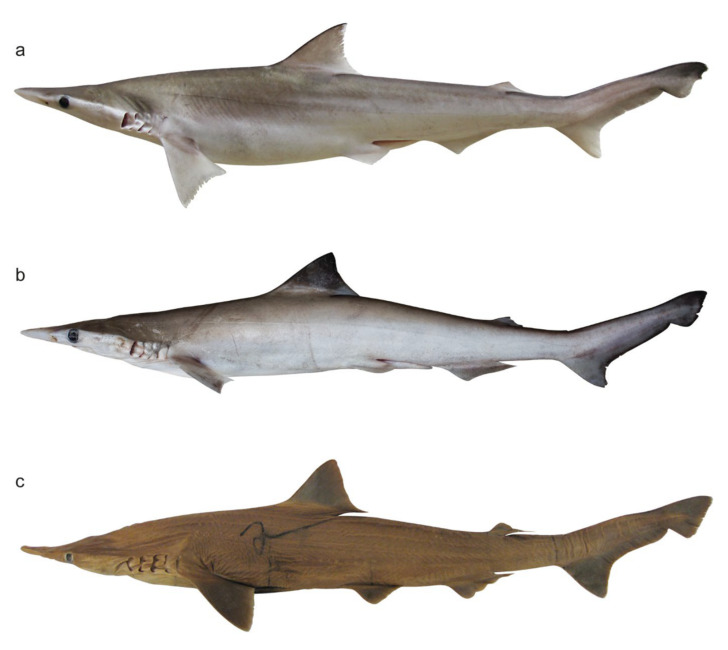
Lateral view of *Scoliodon* ‘species’. (**a**) *S. macrorhynchos* IPPS WWPLAL#1 (adult male 426 mm TL, fresh), (**b**) *S.* cf. *laticaudus* CSIRO H 8401-09 (adult male 394 mm TL), and (**c**) *S. laticaudus* MNHN 1123 (female 524 mm TL, preserved).

A total of 83 morphometric measurements were obtained from 74 *Scoliodon* specimens from a range of locations encompassing a large proportion of the geographic range of the three ‘species’ types: *S. laticaudus*, *S.* cf. *laticaudus*, and *S. macrorhynchos* (Figure 1). A total of 8 specimens of *S. laticaudus* (India); 32 specimens of *S.* cf. *laticaudus* (including the *S. muelleri* holotype from ‘Bengal’, Malacca Strait, and the Ranong fishing port in the Andaman Sea); and 34 specimens of *S. macrorhynchos* (Hong Kong, Indonesia, Borneo, and Taiwan) were measured in full (Table 2). Vertebral counts were taken from radiographs of 13 specimens of *S.* cf. *laticaudus* and 13 specimens of *S. macrorhynchos*. Counts were obtained separately for the trunk (monospondylous), precaudal (monospondylous and diplospondylous to the origin of upper lobe of caudal fin), and caudal (centra of the caudal fin) vertebrae. Tooth row counts were taken in situ or from excised jaws of 7 specimens of *S. laticaudus*, 5 specimens of *S*. cf. *laticaudus*, and 8 specimens of *S. macrorhynchos*.

### 2.5. Multivariate Analyses

Morphometric measurements, as % total length (TL), were subjected to nonmetric multidimensional scaling (MDS) ordination (Primer v 7.0 package, Quest Research Limited, Auckland, New Zealand) to determine whether significant differences between putative species exist or whether intraspecific variations of a single species is a factor. One-way Analyses of Similarity (ANOSIM) were employed to test whether morphometric measurements differed significantly among size classes. Similarity Percentages (SIMPER) were employed when a pairwise ANOSIM result was significant at *p* < 0.05 to determine what characters contributed most to the observed differences. To determine if significant differences between size classes exist, samples were allocated to one of four arbitrary size classes: (1) <249 mm TL, (2) 250–299 mm TL, (3) 300–399 mm TL, and (4) >400 mm TL. Morphometric measurements were analyzed without transformation since the preliminary analyses revealed that the stress levels were acceptable (i.e., <0.3) for MDS analyses (see Clarke and Gorley [46]). Several measurements, associated with the clasper and trunk and abdomen heights and widths, were not available for measurement for all individuals, so these characters were excluded from the MDS analysis.

### 2.6. Museum Holdings

Collection details for the 74 *Scoliodon* specimens examined are provided in Supplementary Data S1. Specimens are referred to by the following prefixes for their registration numbers: BMNH, British Museum of Natural History, London; IPPS, Sarawak Fisheries Research Institute, Bintawa, Malaysia; CSIRO, Australian National Fish Collection, Hobart; RMNH, Rikjsmuseum van Natuurlkjke Histoire, Leiden; and MNHN, Museum National d’Histoire Naturelle, Paris, France.

## 3. Results

### 3.1. Molecular Analysis

**Figure 2:**
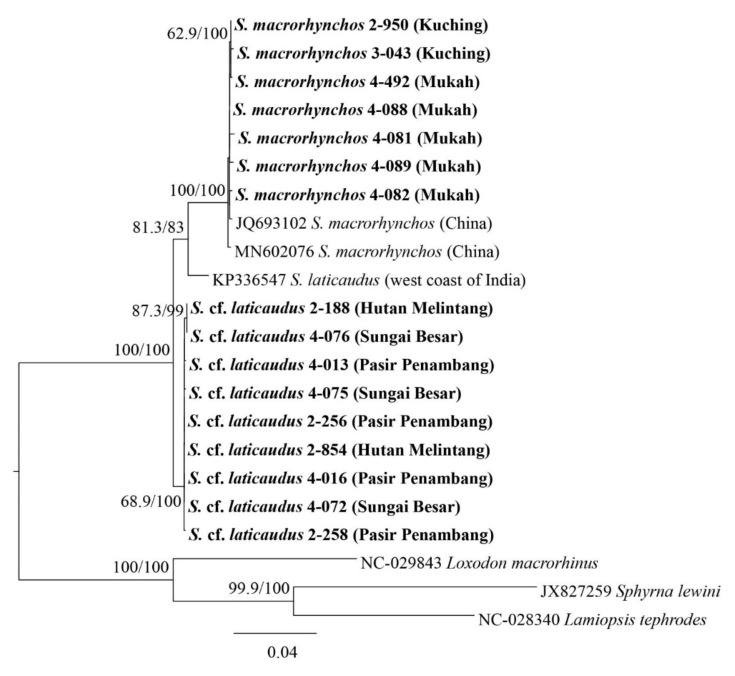
NADH2COI gene mid-point rooting phylogenetic relationships of *Scoliodon* ‘species’ (phylogram). The bootstrap values (ML/BI) are shown at branches. Sequence names in bold are from the present study.

Using the *NADH2* and *COI* mitochondrial DNA sequences (Figure 2 and Supplementary Figure S1a,b), three monophyletic groups with moderate-to-full support bootstrap values (ML 58.3—100/BI 68—100) were identified based on sampling locations, i.e., *Scoliodon laticaudus* from the Indian Ocean (based on samples from the west coast of India), *Scoliodon macrorhynchos* from South China Sea (Kuching and Mukah, both localities in Sarawak, which were grouped with samples from China and Taiwan), and a possible third species from the Malacca Strait, tentatively labeled as *S.* cf. *laticaudus*, were grouped with samples from Bangladesh, Myanmar, and Thailand. The uncorrected p-distances among these three monophyletic groups ranged from 0.61 to 3.06% for *COI*, 2.98 to 4.23% for *NADH2*, and 2.12 to 3.19% for the combined mitochondrial DNA (Table 3).

**Figure 3:**
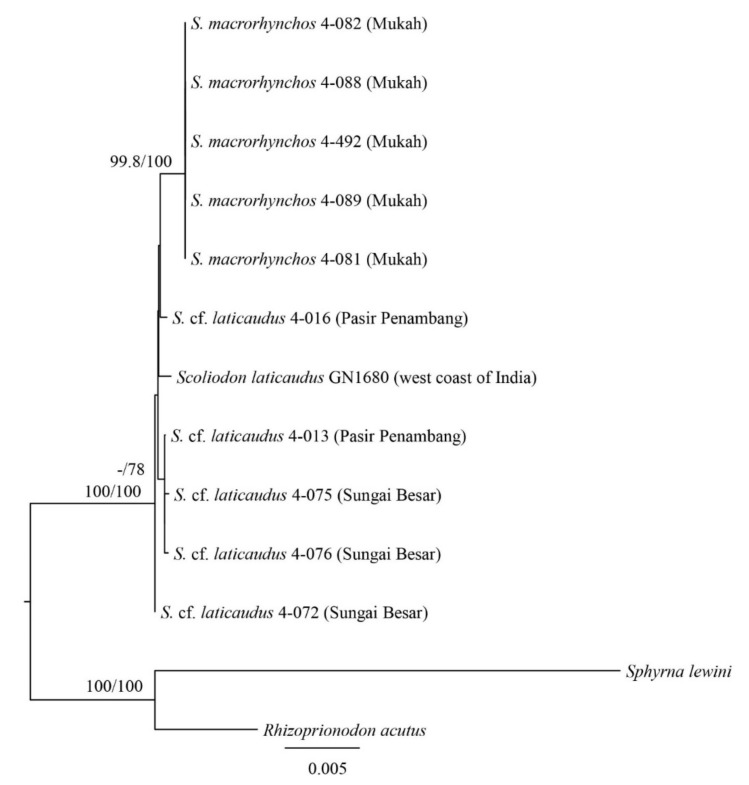
Nuclear gene mid-point rooting phylogenetic relationships of *Scoliodon* ‘species’ (phylogram). The bootstrap values (ML/BI) are shown at branches.

The estimated trees for *Scoliodon* species using nuclear DNA (Figure 2 and Figure 3 and Supplementary Figure S1) showed partial agreement with those using mitochondrial DNA. Three out of five individual nuclear DNA gene trees indicated monophyly of the *Scoliodon* genus (*Prox1*, *RAG1*, and *TOB1*) (Supplementary Figure S1c–i). Topologies of concatenated nuclear DNA estimated tree showed two monophyletic groups, *S. macrorhynchos* and *S. laticaudus*–*S.* cf. *laticaudus* groups (Figure 3). The uncorrected p-distance for nuclear DNA among the three monophyletic groups identified from mitochondrial DNA ranged from 0 to 0.91% (mean 0.2%) (Table 3).

**Figure 4:**
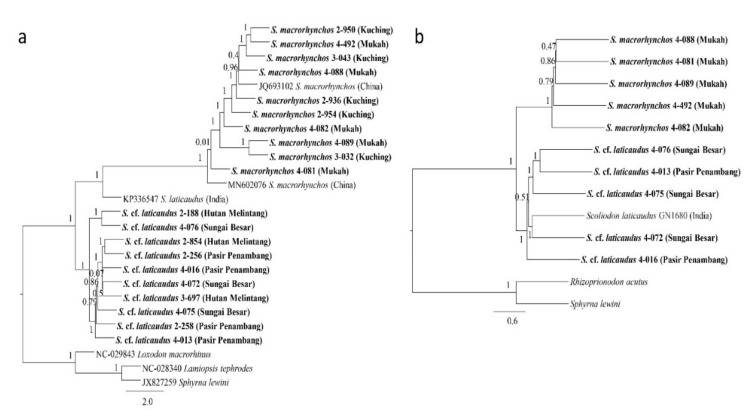
ASTRAL species tree of *Scoliodon* species for (**a**) mtDNA and (**b**) nuclear DNA.

The species tree estimated in ASTRAL for both mitochondrial and nuclear DNA were topologically congruent with their respective gene trees and had a normalized quartet score of 0.81 and 0.61, respectively (Figure 4). The BPP run supported both estimations from traditional phylogenetic analyses (Bayesian and ML) and ASTRAL. Specifically, the BPP run on mtDNA supported the separation of *Scoliodon* into three separate species with a probability of 1 under any combination of the theta and tau priors. The BPP run on nuclear DNA, on the other hand, varied depending on the theta and tau prior settings; settings of theta at 0.1 regardless of tau prior supported the monospecificity of *Scoliodon* (probability > 0.99), theta at 0.001 in combinations with tau at 0.01 and at 0.001 supported separation into three species (probability 0.65–0.88), and other settings in between supported the combination of *S. laticaudus* and *S.* cf. *laticaudus* as a separate group from *S. macrorhynchos* (probability 0.51–0.61).

### 3.2. Morphology and Meristics

No nonoverlapping morphometric ranges were found between the three putative ‘species’ of *Scoliodon*. Likewise, vertebral counts strongly overlapped between the three ‘species’. No dental morphological differences were detected between the three *Scoliodon* ‘species’.

**Figure 5:**
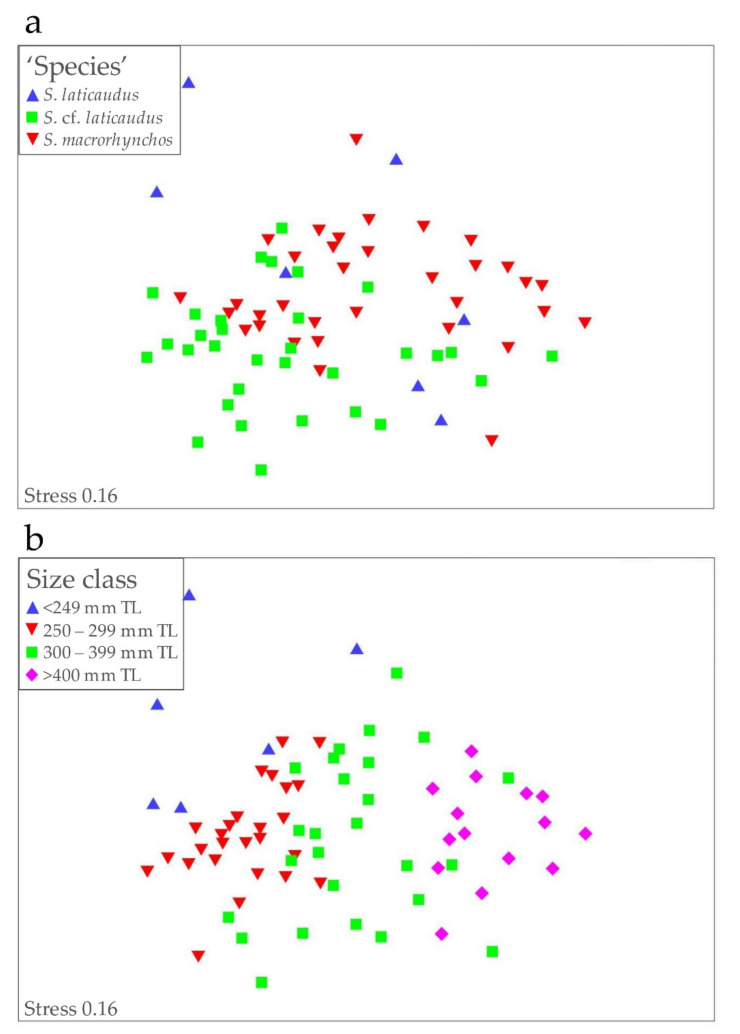
Nonmetric multidimensional scaling (MDS) ordination of *Scoliodon* ‘species’ morphometric percentages (% TL): (**a**) coded by species and (**b**) coded by size class.

The MDS analysis of the measured *Scoliodon* specimens showed considerable overlaps among the three ‘species’ (Figure 5a). Measurements of the limited *S. laticaudus* samples were highly variable but generally fell within the two overlapping clusters of *S. macrorhynchos* and *S.* cf. *laticaudus* animals. ANOSIM showed that the ‘species’ were significantly different overall (*p* < 0.01) although the global R2 value was very low (0.24). Similarly, pairwise comparisons between the three ‘species’ were also significantly different (*p* < 0.01) but with low R2 values (0.18–0.42).

When the same ordination plot was coded by size class (1 ≤ 250 mm TL, 2 = 250–299 mm TL, 3 = 300–399 mm TL, and 4 ≥ 400 mm TL), the samples for each size class formed only partially overlapping groups, with the smallest specimens to the left of the plot and the largest to the right (Figure 5b). ANOSIM showed that the size classes significantly different overall (*p* < 0.01), and with a higher global R2 value (0.54). All pairwise comparisons of size classes were also significantly different (*p* < 0.01), with generally higher R2 values (0.3–0.96). The measurements shown by SIMPER to be the most responsible for the differences between the size classes were pre-anal length, pectoral–pelvic space, pre-pectoral length, pre-pelvic length, head length, and pre-first dorsal length.

## 4. Discussion

Based on a combination of nuclear and mitochondrial markers, the evidence supports the split proposed by White et al. [5]. Evidence from mtDNA suggests genetic isolation among the three ‘species’ types; *S. laticaudus* from India is a separate species from *S. macrorhynchos* from Sarawak, Malaysian Borneo that appears to cluster with samples from China and Taiwan. Evidence from the pooled nuclear markers group *S.* cf. *laticaudus* (from Malacca Strait) with *S. laticaudus*. Both molecular and morphological data presented suggest that any differences that exist among the species of *Scoliodon* are consistent with isolation by distance. We found no evidence of sympatry among any of the three ‘species’. Presently, we cautiously recommend that *S.* cf. *laticaudus* of the Malacca Strait be assigned as *S. laticaudus*. These results and the updated distributional range of the *Scoliodon* species are discussed below.

### 4.1. Taxonomic Conclusions and Recommendations

The decision to resurrect *S. macrorhynchos* as distinct from *S. laticaudus* was primarily based on the *NADH2* sequence data obtained in White et al. [5]. Recent studies have highlighted that the use of single mitochondrial markers alone to distinguish between species can be questionable, especially in light of discordant species trees using mitochondrial and nuclear DNA (for example, *Chimaera ogilbyi* in Finucci et al. [47], freshwater snail genus *Cipangopaludina* in Hirano et al. [48], and terrapins (family Emydidae) in Wiens et al. [49]). In the case of *Scoliodon*, there is considerable concordance between mitochondrial and nuclear signals to support the conclusion of White et al. [5], i.e., the resurrection of *S. macrorhynchos* as a valid species and separate from *S. laticaudus* from India.

Phylogenetic and species trees using combined mitochondrial markers group *S. macrorhynchos* from Sarawak Borneo and from China together, but the same cannot be said for nuclear markers due to the nonavailability of China sequences. Both mitochondrial and nuclear phylogenetic trees mostly support *S. macrorhynchos* from Sarawak Borneo as separate from *S.* cf. *laticaudus* from the Malacca Strait. The discordance between mitochondrial and nuclear signals arises regarding the relationship of *S.* cf. *laticaudus* and *S. laticaudus*. Ambiguity in individual nuclear signals underscores the need to use multiple genes to infer species relationship, and concatenated nuclear signals provisionally group *Scoliodon* individuals from the Malacca Strait as *S. laticaudus*. In addition to congruence between mitochondrial and nuclear data, congruence between molecular and morphological characteristics has also been employed to delimit species (e.g., Finucci et al. [47] and Petean et al. [14]). For *Scoliodon*, White et al. [5] documented only mean differences in several morphometric characteristics but with ranges partially overlapping, i.e., head length, pre-pectoral length, lower labial furrow length, and second dorsal fin origin to anal fin origin. The more comprehensive morphological data presented in this study did not find any nonoverlapping morphological differences in the *Scoliodon* specimens examined. However, given the high intraspecific variability in measurements from *S. laticaudus* (Figure 5a), measurements from additional individuals across a broad distribution range are important to clarify the morphological distinctions between *S. laticaudus* and *S. macrorhynchos*.

The available molecular evidence delimits the Malacca Strait as the easternmost boundary for the range of *S. laticaudus*, thereby extending the distribution of the species based on the most recent International Union for Conservation of Nature (IUCN) assessment [10]. The Malay Peninsula appears to serve as a contemporary physical barrier between the two species. This pattern has been seen for a number of coastal-associated species with limited dispersal abilities, such as bamboosharks [11], guitarfishes [50], groupers [51], sea snails [52], and a number of mangrove species [53]. The molecular differences between morphologically similar but non-sympatric *S. macrorhynchos* and *S. laticaudus* suggest a relatively recent divergence due to geographical isolation with limited mixing that drove allopatric speciation, which is feasible given the complexity of the past geological history of the Sundaland region [54]. Further population genetic studies to corroborate this will help shed light on the evolutionary history and biogeography of the species.

Another important aspect to investigate for *Scoliodon* is the population genetic structure. *Scoliodon* is one of the top landed sharks in terms of both abundance and biomass in surveyed areas within Malaysia [55,56]. A strong coastal affiliation [7] and limited dispersal due to small size are traits that likely promote genetic differentiation and, thus, increase their vulnerability to localized fishing impacts. A similar pattern of a fine-scale population structure has been revealed for a similar small-sized benthic coastal shark, *Chiloscyllium punctatum*, that is subject to high fishing pressure in the Southeast Asian region [11,57]. Further investigation into the genetic structure of *Scoliodon* in Southeast Asia and Indian waters is warranted given the high fishing pressure exerted [58].

### 4.2. Geographic Range

**Figure 6:**
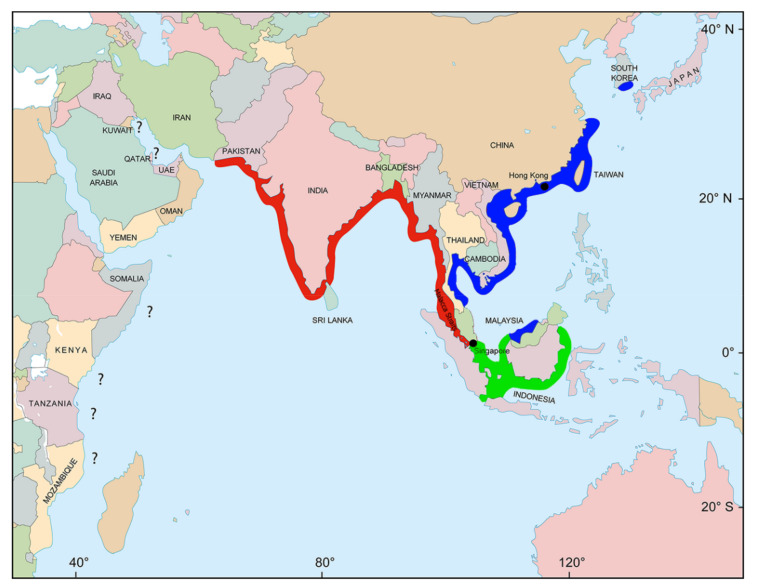
Map of the Indo-West Pacific region showing the refined range of *Scoliodon* species based on the materials examined and a critical examination of the literature. Dubious range locations are highlighted with a question mark. Red = *S. laticaudus*, blue = *S. macrorhynchos*, and green = *Scoliodon* sp. (verification needed).

Distributional ranges for species are often based on a combination of literature sources and expert opinions; therefore, validating some occurrences can be difficult. Since *Scoliodon* is herein confirmed with two valid species, notwithstanding the possibility of another in the Bay of Bengal, it is important to critically investigate the full distributional range for *S. laticaudus* and *S. macrorhynchos*. The identity of *Scoliodon* at locations without genetic sequences is putatively assigned as either *S.* cf. *laticaudus* or *S.* cf. *macrorhynchos* using the Malay Peninsula as the genus distribution break. The resulting distributional range is displayed in Figure 6, with questionable occurrences noted. Investigation of the range is discussed below in an east to west direction.

Off Japan, *S.* cf. *macrorhynchos* has been recorded as a rare occurrence from Kochi Prefecture [59] (as *S. sorrakowah*). Although listed as occurring off the Pacific coast of Southern Japan by Nakaya [60] and Nakabo [61], it is noticeably absent from checklists of coastal fishes in prefectures on the Pacific coast of Southern Japan, e.g., Mie [62], Kagoshima [63], and Nagasaki [64]. Furthermore, nine specimens of *Scoliodon* deposited in Japanese collections with geographic data were caught in either China, Taiwan, or Vietnam (via http://science-net.kahaku.go.jp/, accessed on 28 February 2022). The distribution off Southern Japan appears to be erroneous and should not be included in the range of this species. It has not been previously recorded from South Korea, but Cho et al. [65] reported on a single specimen collected from a Yeosu fish market, Busan in 1995 identified as *S. laticaudus* and supposedly caught from the South Sea of Korea. Off China, Wang [66] noted that *S. macrorhynchos* was abundant off Wenzhou in Southern Zhejiang Province in late spring and early summer but rarely caught in the northern part of the province. Zhu et al. [67] also recorded *S. macrorhynchos* (identified as *S. laticaudus*) from Zhejiang Province but throughout much of the year. Lam and Sadovy de Micheson [68] found that *Scoliodon*, identified as *S. laticaudus*, was the most abundant shark species present during comprehensive market surveys off the Fujian, Hainan, and Guangdong Provinces of China, as well as off Hong Kong. Likewise, Ebert et al. [69] noted that this species was very abundant in fisheries catches around Taiwan.

Naylor et al. [8] provided numerous *NADH2* sequences from specimens caught off Vietnam recorded during local ichthyofaunal surveys. Orlov [70] listed *Scoliodon* spp. as one of the pelagic predators found in marine waters off Cambodia, which likely refers wholly or in part to *S.* cf. *macrorhynchos*. Deechum [71] and Springer [72] included records of *Scoliodon* (identified as *S. laticaudus*) from the Gulf of Thailand. No *Scoliodon* individuals were recorded during comprehensive ichthyofaunal surveys along the east coast of Peninsular Malaysia ([56] Lim et al., unpublished data) but are caught in high abundance in the waters of the west coast of Peninsular Malaysia. As verified by Compagno et al. [73], *Scoliodon* was largely absent in the Philippines. A recent listing of this species in the Philippines elasmobranch identification guide by Alava et al. [74] was likely based on an old record of misidentified *Loxodon* or *Rhizoprionodon*. In Malaysian Borneo, none were recorded from off Sabah from multiple fish surveys, but *S. macrorhynchos* is caught in high abundance off Sarawak ([75] Lim et al., unpublished data, and Manjaji-Matsumoto pers. comm.). *Scoliodon* was not recorded in shark catches off Bintan Island in the Riau Archipelago of Indonesia just to the southeast of Singapore [76].

In Indonesia, *S.* cf. *macrorhynchos* appears to be restricted to Kalimantan [75] and around the river outflows of Eastern Sumatra that flow into the Malacca Strait [77]. It has not been recorded in the literature from West Sumatra or from recent landing site surveys (Fahmi, pers. comm.). Although Bleeker [6] described *S. macrorhynchos* from a juvenile specimen from off Batavia (= Jakarta), which would have likely been caught locally, it has not been recorded off Java in surveys over the last half a century (e.g., Widodo et al. [78] and Widodo and Mahiswara [79]). Springer [72] also listed a specimen deposited at the Smithsonian Institute (USNM 72479) from Batavia (= Jakarta, West Java). This specimen was collected by Owen Bryant and William Palmer in 1909 during a natural history specimen collection trip [80]. Despite being the most abundant species found in recent surveys of the Muara Baru fishing port in Jakarta [81], these were caught in South Kalimantan and only landed in Jakarta. Due to the lack of accurate baseline information, it is not possible to determine whether *Scoliodon* has been extirpated from Javan waters due to overexploitation.

Arunrugstichai et al. [26] recorded *S. laticaudus* as one of the most abundant shark species landed off the Andaman Coast of Thailand. Psomadakis et al. [82] stated that this species is found in coastal waters and lower reaches of the rivers in Myanmar. Jit et al. [83] recorded it as the most abundant shark species based on surveys of two landing centers in Bangladesh, i.e., Chittagong and Cox’s Bazar. *Scoliodon laticaudus* is abundant off the Indian coastline, with verified records from all coastal states (from east to west): Andaman and Nicobar archipelago [84,85], West Bengal [86], Orissa [87,88], Andhra Pradesh [89], Tamil Nadu [90], Kerala [91], Karnataka [92,93], Goa [94], Maharashtra [95,96], and Gujarat [97]. *Scoliodon laticaudus* has not been recorded from the Indian union territory of Lakshadweep (formerly Laccadive Archipelago) nor further south in the Maldives or Chagos Archipelago. The presence of *S. laticaudus* off Sri Lanka is less clear. Some checklists have included this species from Sri Lankan waters, e.g., Misra [98] (as *Scoliodon sorrakowah*), Mendis [99] (as *Carcharias laticaudus*), and De Silva [100]. However, recent surveys of 15 fish markets around Sri Lanka recorded no *Scoliodon* [101]. Likewise, Moron et al. [102] did not include this species as present off the west coast of Sri Lanka. Given that *Scoliodon* is usually found in abundance where it occurs, its absence is notable in these studies. Thus, it may be absent from Sri Lankan waters or restricted to only the northern part of Sri Lanka around Palk Bay and the Gulf of Mannar, where it is known to be abundant on the respective Indian coastlines. Off Pakistan, *S.* cf. *laticaudus* was recorded from the coasts of the Sindh Province (Misra [103] as *S. sorrakowah*) and a single specimen recorded during port surveys at Jiwani in Westernmost Balochistan Province, close to the Iranian border [104].

The range of *Scoliodon* has recently included the Persian Gulf and parts of East Africa [7,9]. Bishop [105] and Sivasubramanian and Ibrahim [106] recorded it from off Kuwait and Qatar, respectively, but more recent comprehensive surveys of these locations, as well as of Bahrain and the United Arab Emirates, did not record any *S. laticaudus* in fisheries landings [107,108,109]. Amojil et al. [110] included this species as only possibly occurring in the Persian Gulf due to the lack of verifiable records. *Scoliodon* cf. *laticaudus* was not recorded during comprehensive surveys of fish landing sites in Oman [111,112]. It was also not recorded from catches of Russian trawlers operating off the entire Yemen coast (including Socotra Island) between 1985 and 1990 [113] or in a recent comprehensive survey of the fish fauna of Socotra Islands [114].

*Scoliodon* cf. *laticaudus* was included as part of the marine fauna of Somalia [115] and reported as rare in the Somali shark fishery [116]. Although included in a species catalog of Kenya [117], surveys of catches in small-scale fisheries off Kenya over the last decade have not recorded any individuals of this species ([118] B. Kiilu, pers. comm.). Compagno [4] included Tanzania in the range for *S.* cf. *laticaudus* and also included it as present in Mozambique [119]. However, this species has not been recorded from fishery bycatches in recent years in either Mozambique or Tanzania (A. Marshall, S. Pierce, C. Rohner, and D. Ebert, pers. comm.). The presence of *Scoliodon* in the fauna of East Africa from Somalia to Mozambique is dubious. Where *S. laticaudus* is found, they are typically caught in high numbers and common in coastal waters. It is more likely that they are misidentifications of similar species, e.g., *Rhizoprionodon acutus*, which was previously referred to as *Scoliodon walbeehmi* throughout the Indo-West Pacific before being synonymized. Thus, the East Africa distribution of *S. laticaudus* is treated as dubious.

The present distribution delineation is mostly consistent with the recently published IUCN assessment for *S. laticaudus* [10] and *S. macrorhynchos* [120]. In a largely contiguous coastline distribution of *Scoliodon* (Figure 6), we noted two contemporary spatial ‘breaks’, i.e., along the east coast of the Malaysian Peninsula and off the Sabah coastline of Northeastern Borneo. These breaks could be due to the presence of unsuitable bottom habitats for the species (Manjaji-Matsumoto, pers. comm.) and also reflect the complex evolutionary history of the Sundaland region. Notably, the presence and taxonomic status of *Scoliodon* in the Indonesian region, especially along Eastern Sumatra and along the Kalimantan coastline (Figure 6), needs to be investigated using an integrative approach, i.e., molecular and morphological analyses. It was hypothesized that animals along Eastern Sumatra are *S. laticaudus*, while those in Kalimantan waters are *S. macrorhynchos*, with the Karimata Strait acting as a physical and/or genetic barrier—this is consistent with evidence presented for the genetic structure seen for *C. punctatum* [11].

## 5. Conclusions

Collective evidence from mitochondrial DNA, nuclear DNA, and morphological analyses clearly supports the previous resurrection of *S. macrorhynchos* as distinct species from *S. laticaudus*. Genetic distinctiveness between the two species is likely a product of isolation by distance with the Malaysian Peninsula acting as a physical barrier. The identity of *Scoliodon* from Indonesian waters remained unverified and should be the focus for future taxonomic studies. Both *Scoliodon* species are currently classified as “near threatened” in the IUCN Red List. With the new evidence from this study, we recommend updating the distribution information of these species and investigating the taxonomic status of *Scoliodon* animals from Indonesian coastal waters.

## Figures and Tables

**Table 1:** Primers used in this study and their references.

Marker	Forward Primer (5′–3′)	Reverse Primer (5′–3′)	References
*COI*	FishF2—TCG ACT AAT CAT AAA GAT ATC GGC AC	FishR2—ACT TCA GGG TGA CCG AAG AAT CAG AA	Ward et al. [29]
*NADH2*	ILEM—AAG GAG CAG TTT GAT AGA GT	ASNM—AAC GCT TAG CTG TTA ATT AA	Naylor et al. [30]
*ACT*	ACT-F—GCT TTC ATC TCC TTC GGC AGT TTG	ACT-R—CCA CTG GTA ATT GGG ATA CTT GGC	Design based on GN’s sequence of sample GN1680
*KBTBD2*	KBT-F—CTC AGT ATC TAT CTT CAG TCC TTG GC	KBT-R—GCT CTT ACA CAG GGA TCA GAG TAG C	Design based on GN’s sequence of sample GN1680
*PROX1*	PRO1-F—AAT TCT TCA AGG GAA AGT GCC CAA G	PRO1-R—CAG ACT GCT CCG ACG AGT TTT TG	Design based on GN’s sequence of sample GN1680
*RAG1*	RAG1-F—CTT ATT CAA ACC ATC AAC AAC ACA ACA	RAG1-R—CTG CAT GAC TGC TTC CAA CTC ATC	Design based on GN’s sequence of sample GN1680
*RAG2*	RAG2-F—TCA GAA TCA AAC AGC CTC ATT TAC C	RAG2-R—TTA ATT TCA TTG GAC CAT TCT GGG G	Design based on GN’s sequence of sample GN1680
*SCFD2*	SCFD-F—AGG TGA AAG CGG TAT TTG TGG TG	SCFD-R—TGA GCT GCA GAA CTT CAA ACA TAG	Design based on GN’s sequence of sample GN1680
*TOB1*	TOB1-F—ATA TGA AGG TCA CTG GTA TCC AGA C	TOB1-R—GAA AAC AAA CTC CTT GGC ATT GGG A	Design based on GN’s sequence of sample GN1680

**Table 2:** Ranges of proportional dimensions as percentages of the total length for the three ‘species’ of *Scoliodon*.

	*S. laticaudus*		*S.* cf. *laticaudus*		*S. macrorhynchos*	
	*n* = 8		*n* = 32		*n* = 34	
	Min.	Max.	Min.	Max.	Min.	Max.
Total length (mm)	169	524	239	490	227	562
Precaudal length	75.6	78.0	75.3	78.0	73.6	78.0
Pre-second dorsal length	62.6	65.4	62.9	66.7	61.5	66.5
Pre-first dorsal length	35.1	38.8	33.0	37.7	33.0	38.1
Head length	21.5	29.1	21.5	26.3	21.3	25.6
Head length (horiz)	21.0	28.6	21.0	25.1	20.9	25.0
Pre-branchial length	17.1	23.5	17.1	20.4	16.5	20.7
Pre-branchial length (horiz)	16.6	22.6	16.5	19.8	16.0	19.5
Preorbital length	8.9	12.6	8.9	11.7	8.5	11.6
Preorbital length (horiz)	8.1	11.3	7.9	10.8	7.0	10.7
Preoral length	7.1	11.1	7.1	10.4	7.2	10.4
Pre-narial length	6.6	9.1	6.6	8.7	6.2	8.4
Pre-narial length (horiz)	5.9	8.2	5.6	8.1	4.8	7.8
Pre-pectoral length	22.1	26.4	21.5	26.6	20.1	26.2
Pre-pelvic length	43.9	48.4	43.9	50.2	43.8	49.2
Snout–vent length	45.9	49.2	45.9	51.4	45.4	50.6
Preanal length	56.7	59.9	56.7	62.0	54.8	60.4
Interdorsal space	16.1	21.7	17.9	21.7	17.9	22.2
Dorsal-caudal space	7.2	9.3	7.2	9.9	7.2	9.4
Pectoral–pelvic space	16.7	19.7	16.9	20.7	16.8	21.6
Pelvic–anal space	5.2	9.0	5.6	11.1	4.8	8.7
Anal–caudal space	6.4	9.1	6.4	8.8	6.4	9.1
Eye length	1.5	2.2	1.6	2.5	1.3	2.4
Eye height	1.3	2.5	1.3	2.5	1.5	2.2
Interorbital space	7.4	11.2	7.4	9.8	7.5	10.3
Nostril width	1.4	2.0	1.5	2.3	1.4	2.3
Internarial space	4.9	6.9	4.9	6.7	4.9	6.5
Anterior nasal flap length	0.2	0.6	0.2	0.5	0.2	0.6
Mouth length	4.5	5.6	4.1	4.9	3.5	5.2
Mouth width	6.0	7.6	5.3	7.6	5.7	7.6
Upper labial furrow length	0.2	0.6	0.1	0.5	0.1	0.5
Lower labial furrow length	0.8	1.2	0.2	1.5	0.3	1.4
First gill slit height	2.3	3.1	2.3	4.1	2.2	4.0
Second gill slit height	2.3	3.6	2.1	2.6	2.2	3.2
Third gill slit height	2.4	3.8	2.2	4.7	2.3	4.4
Fourth gill slit height	2.4	3.7	2.0	2.8	2.4	3.3
Fifth gill slit height	2.2	3.2	2.1	3.3	2.3	3.1
Intergill length	4.6	5.9	4.6	5.4	4.5	6.4
Head height	6.1	10.2	7.7	9.9	7.0	10.6
Trunk height	7.9	10.8	8.3	10.8	7.8	13.1
Abdomen height	7.5	11.2	10.0	11.4	9.4	13.9
Tail height	6.3	10.2	7.0	9.4	7.5	11.3
Caudal peduncle height	3.9	4.5	3.8	4.5	4.0	5.0
Head width	7.3	9.4	6.9	9.9	7.9	10.8
Trunk width	6.4	8.5	6.5	8.8	6.2	11.8
Abdomen width	5.2	7.1	4.9	6.9	5.2	8.9
Tail width	4.1	5.6	4.2	5.6	4.6	6.5
Caudal peduncle width	1.9	2.7	2.3	3.5	2.2	3.7
Pectoral length	10.2	12.1	9.8	11.6	9.9	11.7
Pectoral anterior margin	9.5	12.1	9.4	11.5	9.2	11.9
Pectoral base	4.5	6.6	5.2	6.4	4.8	6.6
Pectoral height	7.8	10.3	7.4	10.3	7.5	10.1
Pectoral inner margin	5.2	6.2	4.6	6.4	4.3	6.2
Pectoral posterior margin	6.3	10.6	6.8	12.5	6.8	9.8
Pelvic length	7.3	8.7	7.1	8.9	6.9	8.3
Pelvic anterior margin	4.7	5.4	4.3	6.0	4.3	5.6
Pelvic base	4.7	5.6	4.3	6.3	4.3	6.1
Pelvic height	3.2	4.3	2.3	4.4	2.7	4.2
Pelvic inner margin length	2.2	3.7	2.1	3.9	2.2	3.5
Pelvic posterior margin length	3.4	5.3	3.4	5.3	3.8	5.1
Clasper outer length	6.0	9.0	4.5	10.2	4.0	10.0
Clasper inner length	8.4	11.8	6.4	12.4	6.5	12.1
Clasper base width	1.0	1.4	0.6	1.7	0.6	1.4
First dorsal length	13.3	15.6	13.3	15.7	12.9	15.5
First dorsal anterior margin	11.1	13.5	11.8	14.3	11.2	14.6
First dorsal base	8.9	10.9	8.9	11.4	8.8	11.0
First dorsal height	6.6	8.6	5.8	8.8	6.5	9.0
First dorsal inner margin	3.8	5.1	3.9	5.3	3.5	4.9
First dorsal posterior margin	6.7	9.2	5.7	9.0	6.2	8.9
Second dorsal length	7.5	9.3	7.3	9.1	6.9	8.6
Second dorsal Anterior margin	4.1	5.5	3.4	5.5	3.4	5.0
Second dorsal base	4.0	4.8	3.2	4.9	3.2	4.8
Second dorsal height	1.7	2.2	1.2	2.4	1.3	2.0
Second dorsal inner margin	3.2	4.7	3.8	5.1	3.3	4.8
Second dorsal posterior margin	3.8	5.3	3.9	4.9	3.6	4.7
Anal length	11.4	13.5	9.6	13.7	10.8	14.1
Anal anterior margin	5.1	6.7	4.1	7.0	4.9	7.8
Anal base	8.0	10.3	6.1	10.3	7.2	11.2
Anal height	2.8	3.7	2.2	3.6	2.6	3.8
Anal Inner margin	3.0	3.9	3.0	3.9	2.8	4.1
Anal posterior margin	6.6	8.4	5.8	8.8	6.5	8.9
Dorsal caudal margin	22.0	24.9	21.6	24.6	21.9	25.6
Pre-ventral caudal margin	8.5	10.2	7.8	10.7	8.0	10.5
Lower post-ventral caudal margin	3.4	4.7	2.9	5.0	2.9	4.8
Upper post-ventral caudal margin	9.5	11.5	8.9	11.0	9.1	12.3
Caudal fork width	5.4	7.5	5.4	6.8	5.9	7.1
Caudal fork length	7.8	9.7	8.0	9.8	7.8	9.8
Subterminal caudal margin	3.9	5.6	3.1	4.7	3.1	5.3
Subterminal caudal width	2.6	3.4	2.6	3.5	2.7	3.4
Terminal caudal margin	4.5	7.4	4.8	6.8	4.9	7.3
Terminal caudal lobe	7.6	8.9	6.8	8.6	7.2	9.3
Second dorsal origin	4.6	6.9	3.0	6.9	5.2	9.1
Second dorsal insertion	0.5	2.2	0.6	2.0	0.6	2.7
Mid-base first dorsal fin to pectoral insertion	10.9	12.7	10.5	13.4	11.0	14.6
Mid-base first dorsal fin to pelvic origin	4.4	6.2	4.6	7.9	4.4	7.6
First dorsal insertion to pelvic mid-base	2.8	3.9	2.8	5.4	1.9	5.1
Pelvic mid-base to second dorsal origin	12.9	18.1	13.6	18.1	13.5	19.0

**Table 3:** Genetic distance range (mean, in percent) among monophyletic groups in mitochondrial DNA and nuclear DNA phylogenetic trees. Slat—*Scoliodon laticaudus*, Scflat—*S*. cf. *laticaudus*, and Smac—*S. macrorhynchos*.

	Slat-Scflat	Slat-Smac	Scflat-Smac
COI	0.82 (0.61–1.53)	2.35 (1.99–2.75)	2.29 (2.14–3.06)
NADH2	3.05 (2.98–3.27)	3.06 (2.98–3.26)	3.64 (3.46–4.23)
Mitochondrial	2.16 (2.12–2.18)	2.82 (2.71–2.89)	3.05 (2.95–3.18)
ACT	0.10 (0.00–0.25)	0.50 (0.50–0.50)	0.50 (0.25–0.74)
KBTBD2	0.00 (0.00–0.00)	0.22 (0.22–0.22)	0.22 (0.22–0.22)
PROX1	0.00 (0.00–0.00)	0.02 (0.00–0.11)	0.02 (0.00–0.11)
RAG1	0.12 (0.12–0.12)	0.12 (0.12–0.12)	0.02 (0.00–0.12)
RAG2	0.54 (0.45–0.61)	0.91 (0.91–0.91)	0.58 (0.45–0.61)
SCFD2	0.13 (0.00–0.21)	0.21 (0.21–0.21)	0.17 (0.00–0.42)
TOB1	0.00 (0.00–0.00)	0.00 (0.00–0.00)	0.00 (0.00–0.00)
Nuclear	0.12 (0.10–0.14)	0.25 (0.25–0.25)	0.19 (0.16–0.21)

## Data Availability

Ranges of the morphological data obtained in this study are provided in Table 2. The raw morphological data generated during this current study are available from the corresponding author on reasonable request. All sequences used in this study have been deposited in GenBank, and the related accession numbers are provided in the related figure and text sections.

## Supplementary Materials

The following supporting information can be downloaded at www.mdpi.com/article/10.3390/ani12060681/s1: Table S1: Genetic samples used in this study with locality data and GenBank accession numbers for each of the mitochondrial and nuclear markers. Table S2: Best model selected for maximum likelihood and Bayesian inference analysis according to each marker and the combined markers. Table S3: NCBI GenBank and Barcode of Life Data (BOLD) Systems accession number of the reference sequences used in the analyses. Data S1: Collection data for all specimens of *Scoliodon* examined in this study. Figure S1a: *COI* gene phylogenetic relationships of *Scoliodon* species (phylogram). The bootstrap values (ML/BI) are shown at branches. Sequence names in bold were from the present study. Figure S1b: *NADH2* gene phylogenetic relationships of *Scoliodon* species (phylogram). The bootstrap values (ML/BI) are shown at branches. Sequence names in bold were from the present study. Figure S1c: *ACT* phylogenetic relationships of *Scoliodon* species (phylogram). The bootstrap values (ML/BI) are shown at branches. Figure S1d: *KBTBD2* phylogenetic relationships of *Scoliodon* species (phylogram). The bootstrap values (ML/BI) are shown at branches. Figure S1e: *PROX1* phylogenetic relationships of *Scoliodon* species (phylogram). The bootstrap values (ML/BI) are shown at branches. Figure S1f: *RAG1* phylogenetic relationships of *Scoliodon* species (phylogram). The bootstrap values (ML/BI) are shown at branches. Figure S1g: *RAG2* phylogenetic relationships of *Scoliodon* species (phylogram). The bootstrap values (ML/BI) are shown at branches. Figure S1h: *SCFD1* phylogenetic relationships of *Scoliodon* species (phylogram). The bootstrap values (ML/BI) are shown at branches. Figure S1i: *TOB1* phylogenetic relationships of *Scoliodon* species (phylogram). The bootstrap values (ML/BI) are shown at branches.
