# Supplementary material for: Integrated Taxonomy Revealed Genetic Differences in Morphologically Similar and Non-Sympatric Scoliodon macrorhynchos and S. laticaudus
Source: Animals (Basel). 2022 Mar 8;12(6):681. doi: 10.3390/ani12060681 (PMC8944610; doi:10.3390/ani12060681)
Supplement: Supplementary file 1 [file animals-12-00681-s001.zip › animals-1593741-SI.pdf]

animals

MDPI

Article

Integrated Taxonomy Revealed Genetic Differences in Morphologically Similar and Non-Sympatric *Scoliodon macrorhynchos* and *S. laticaudus*

Kean Chong Lim [1], William T. White [2], Amy Y. H. Then [3,*], Gavin J. P. Naylor [4], Sirachai Arunrugstichai [5] and Kar-Hoe Loh [1,*]

1 Institute of Ocean and Earth Sciences, Universiti Malaya, Kuala Lumpur 50603, Malaysia; keanchonglim@gmail.com
2 CSIRO National Research Collections Australia, Australia National Fish Collection, Hobart, TAS 7001, Australia; william.white@csiro.au
3 Institute of Biological Sciences, Universiti Malaya, Kuala Lumpur 50603, Malaysia
4 Florida Museum of Natural History, Dickinson Hall, Gainesville, FL 32601, USA; gjpnaylor@gmail.com
5 Aow Thai Marine Ecology Centre, Bangkok 10100, Thailand; carcharodon.shinalodon@gmail.com
* Correspondence: amy_then@um.edu.my (A.Y.H.T.); khloh@um.edu.my (K.-H.L.)

Citation: Lim, K.C.; White, W.T.; Then, A.Y.H.; Naylor, G.J.P.; Arunrugstichai, S.; Loh, K.-H. Integrated Taxonomy Revealed Genetic Differences in Morphologically Similar and Non-Sympatric *Scoliodon macrorhynchos* and *S. laticaudus*. *Animals* **2022**, *12*, 681. https://doi.org/10.3390/ani12060681

Academic Editor: Martina Francesca Marongiu

Received: 27 January 2022
Accepted: 5 March 2022
Published: 8 March 2022

Publisher's Note: MDPI stays neutral with regard to jurisdictional claims in published maps and institutional affiliations.

Copyright: © 2022 by the authors. Licensee MDPI, Basel, Switzerland. This article is an open access article distributed under the terms and conditions of the Creative Commons Attribution (CC BY) license (https://creativecommons.org/licenses/by/4.0/).

Table S1 Genetic samples used in this study with locality data and GenBank accession numbers for each of the mitochondrial and nuclear markers.

No.	Species	Sample	Location	Accession No.								
				COI	NADH2	ACT	KBTBD2	PROX1	RAG1	RAG2	SCFD2	TOB1
1.	*S.* cf. *laticaudus*	2-188	Hutan Melintang, Malaysia	MW313850	MW313870							
2.	*S.* cf. *laticaudus*	2-256	Pasir Penambang, Malaysia	MW313851	MW313871							
3.	*S.* cf. *laticaudus*	2-258	Pasir Penambang, Malaysia	MW313852	MW313872							
4.	*S.* cf. *laticaudus*	2-854	Hutan Melintang, Malaysia	MW313853	MW313873							
5.	*S.* cf. *laticaudus*	3-697	Hutan Melintang, Malaysia	MW313859								
6.	*S.* cf. *laticaudus*	4-013	Pasir Penambang, Malaysia	MW313860	MW313876	MW313886	MW313936	MW313916	MW313896	MW313906	MW313946	MW313926
7.	*S.* cf. *laticaudus*	4-016	Pasir Penambang, Malaysia	MW313861	MW313877	MW313887	MW313937	MW313917	MW313897	MW313907	MW313947	MW313927
8.	*S.* cf. *laticaudus*	4-072	Sungai Besar, Malaysia	MW313862	MW313878	MW313888	MW313938	MW313918	MW313898	MW313908	MW313948	MW313928
9.	*S.* cf. *laticaudus*	4-075	Sungai Besar, Malaysia	MW313863	MW313879	MW313889	MW313939	MW313919	MW313899	MW313909	MW313949	MW313929
10.	*S.* cf. *laticaudus*	4-076	Sungai Besar, Malaysia	MW313864	MW313880	MW313890	MW313940	MW313920	MW313900	MW313910	MW313950	MW313930
11.	*S. macrorhynchos*	2-936	Kuching, Malaysia	MW313854								
12.	*S. macrorhynchos*	2-950	Kuching, Malaysia	MW313855	MW313874							
13.	*S. macrorhynchos*	2-954	Kuching, Malaysia	MW313856								
14.	*S. macrorhynchos*	3-032	Kuching, Malaysia	MW313857								
15.	*S. macrorhynchos*	3-043	Kuching, Malaysia	MW313858	MW313875							
16.	*S. macrorhynchos*	4-081	Mukah, Malaysia	MW313865	MW313881	MW313891	MW313941	MW313921	MW313901	MW313911	MW313951	MW313931
17.	*S. macrorhynchos*	4-082	Mukah, Malaysia	MW313866	MW313882	MW313892	MW313942	MW313922	MW313902	MW313912	MW313952	MW313932
18.	*S. macrorhynchos*	4-088	Mukah, Malaysia	MW313867	MW313883	MW313893	MW313943	MW313923	MW313903	MW313913	MW313953	MW313933
19.	*S. macrorhynchos*	4-089	Mukah, Malaysia	MW313868	MW313884	MW313894	MW313944	MW313924	MW313904	MW313914	MW313954	MW313934
20.	*S. macrorhynchos*	4-492	Mukah, Malaysia	MW313869	MW313885	MW313895	MW313945	MW313925	MW313905	MW313915	MW313955	MW313935

Table S2 Best model selected for maximum likelihood and Bayesian inference analysis according to each marker and the combined markers.

Markers	Maximum Likelihood	Bayesian Inference
COI	J2 + Gamma	HKY85 + Gamma
NADH2	J2 + Gamma	J2 + Gamma
**Mitochondrial**	**J2 + Gamma**	**J2 + Gamma**
ACT	HKY85 + Gamma	HKY85 + Gamma
RAG1	HKY85 + Gamma	HKY85
RAG2	HKY85 + Gamma	K80
PROX1	TIM	TIM
TOB1	TN93ef	TN93ef
KBTBD2	J1 + Gamma	HKY85 + Gamma
SCFD2	K80	K80
**Nuclear**	**TIM + Gamma**	**HKY85 + Gamma**

Table S3 NCBI GenBank and Barcode of Life Data (BOLD) Systems accession number of the reference sequences used in the analyses.

**Species**	**COI**	**NADH2**
Lamiopsis tephrodes	NC-028340	NC-028340
Loxodon macrorhinus	NC-029843	NC-029843
Scoliodon cf. *laticaudus*	FAOL400-10, FAOL401-10, FAOL403-10, MH087056, MH230956, MH235708, MH429292, MN458374	JQ518655
Scoliodon laticaudus	ANGBF11072-15, ANGBF11077-15, INELA009-12, INELA011-12, KERRI374-08, KP336547, PHANT755-08, PHANT758-08	JQ518654, KP336547
Scoliodon macrorhynchos	IRREK817-08, JQ693102, KERRI701-08, KERRI711-08, KERRI733-08, KERRI751-08, MN602076, MG220558, MG220559, MG220560	JQ519090, JQ693102, KF927962, KF928963, KF927964, KF927965, MN602076
Sphyrna lewini	JX827259	JX827259

Supplementary Data S1 Collection data for all specimens of *Scoliodon* examined in this study.

Scoliodon laticaudus

India: MNHN 1066 (3 specimens), juvenile males 169, 187 and 258 mm TL, MNHN 1122, adult male 425 mm TL, Malabar coast, India, ~11° N, ~76° E; MNHN 1123, female 524 mm TL, Maharashtra, Mumbai, India, 18°56’ N, 72°51’ E; MNHN 1125, female 342 mm TL, Indian Ocean, exact locality not recorded; RMNH 8574, subadult male 353 mm TL, Malabar coast, India.

Scoliodon cf. *laticaudus*

Andaman Sea: CSIRO H 8400-01, female 478 mm TL, CSIRO H 8400-02, adult male 404 mm TL, Ranong fishing port, Thailand, 20 Jul. 2014; CSIRO H 8401-01, adult male 369 mm TL, CSIRO H 8401-02, female 278 mm TL, CSIRO H 8401-03, adult male 362 mm TL, CSIRO H 8401-04, female 263 mm TL, CSIRO H 8401-05, juvenile male 262 mm TL, CSIRO H 8401-06, female 302 mm TL, CSIRO H 8401-07, female 320 mm TL, CSIRO H 8401-08, female 294 mm TL, CSIRO H 8401-09, adult male 394 mm TL, CSIRO H 8401-10, female 265 mm TL, Ranong fishing port, Thailand, 25 Apr. 2015.

Bay of Bengal: MNHN 1041 (holotype of *S. muelleri*), adult male 490 mm TL, Bengal (now West Bengal in India and Bangladesh).

Peninsular Malaysia: unreg (field code 280416SB11), female 361 mm TL, unreg (field code 280416SB13), male 315 mm TL, unreg (field code 280416SB15), female 318 mm TL, unreg (field code 280416SB16), male 346 mm TL, Sungai Besar landing site, Malacca Strait, Malaysia, 28 Apr. 2016; unreg (field code 230316PPe16), male 273 mm TL, unreg (field code 230316PPe18), male 305 mm TL, unreg (field code 230316PPe19), female 292 mm TL, unreg (field code 230316PPe21), female 281 mm TL, unreg (field code 230316PPe22), male 331 mm TL, unreg (field code 230316PPe27), female 292 mm TL, Pasir Penambang landing site, Malacca Strait, Malaysia, 23 Mar. 2016; not retained specimens: 4 females (239–340 mm TL) and 7 males (253–316 mm TL), Malacca Strait, Malaysia.

Scoliodon macrorhynchos:

Hong Kong: CSIRO H 7074–01, female 325 mm TL, CSIRO H 7074–02, subadult male 337 mm TL, CSIRO H 7074–03, female 353 mm TL, CSIRO H 7074–04, female 346 mm TL, CSIRO H 7076–01, adult male 562 mm TL, CSIRO H 7076–02, pregnant female 511 mm TL

(jaws and chondrocranium only), CSIRO H 7076–03, (2 embryos from CSIRO H 7076–02) females 147 and 148 mm TL, CSIRO H 7076–04, adult male 555 mm TL (jaws and chondrocranium only), CSIRO H 7076–05, female 548 mm TL, Tuen Mun fish market, caught from Pearl River Estuary, Hong Kong, ~22°20' N, 113°50' E, 04 Sept. 2009.

Indonesia: BMNH 1867.11.28.190, female 466 mm TL, probably collected with the lost holotype (Jakarta, Indonesia); CSIRO H 5861–01, adult male 436 mm TL, CSIRO H 5861–05, female ~440 mm TL (tail tip damaged), CSIRO H 5861–06, female 239 mm TL, Muara Baru fish landing site, Jakarta, Indonesia (from southern Kalimantan), 18 Mar. 2002; RMNH 7369 (2), female 426 mm TL, female 236 mm TL, Jakarta (as Batavia), Indonesia, 1851?.

Malaysia: CSIRO H 4425–01, subadult male 317 mm TL, CSIRO H 4425–02, female 359 mm TL, Kuching, Sarawak, Malaysia, 16 Oct. 1995; CSIRO H 6227–01, adult male 387 mm TL, Sarawak, Malaysia, Jun. 2002; IPPS HBO22, adult male 390 mm TL, Mukah, Sarawak, Malaysia, 02°53.52' N, 112°05.44' E, 2002; IPPS WWPLAL#1, adult male 426 mm TL, IPPS WWPLAL#2, female 456 mm TL, IPPS WWPLAL#3, female 396 mm TL, IPPS WWPLAL#4, female 344 mm TL, Kuching fish market, Sarawak, Malaysia, 06 Nov. 2009; unreg (field code 050416MK37), male 264 mm TL, unreg (field code 050416MK38), female 262 mm TL, unreg (field code 050416MK39), male 252 mm TL, unreg (field code 050416MK40), male 299 mm TL, Mukah, Sarawak, Malaysian Borneo, 5 Apr. 2016; unreg (field code 060416MK11), female 303 mm TL, unreg (field code 060416MK12), male 364 mm TL, unreg (field code 060416MK16), female 309 mm TL, unreg (field code 060416MK25), male 286 mm TL, unreg (field code 060416MK26), male 291 mm TL, Mukah, Sarawak, Malaysian Borneo, 6 Apr. 2016; not retained specimens: 3 females (287–309 mm TL) and 3 males (227–290 mm TL), Malacca Strait, Malaysia.

Taiwan: CSIRO H 6295–21, adult male 458 mm TL, Tashi fish landing site, northeastern Taiwan, 24 May 2005.

Figure S1 Supplementary figures.

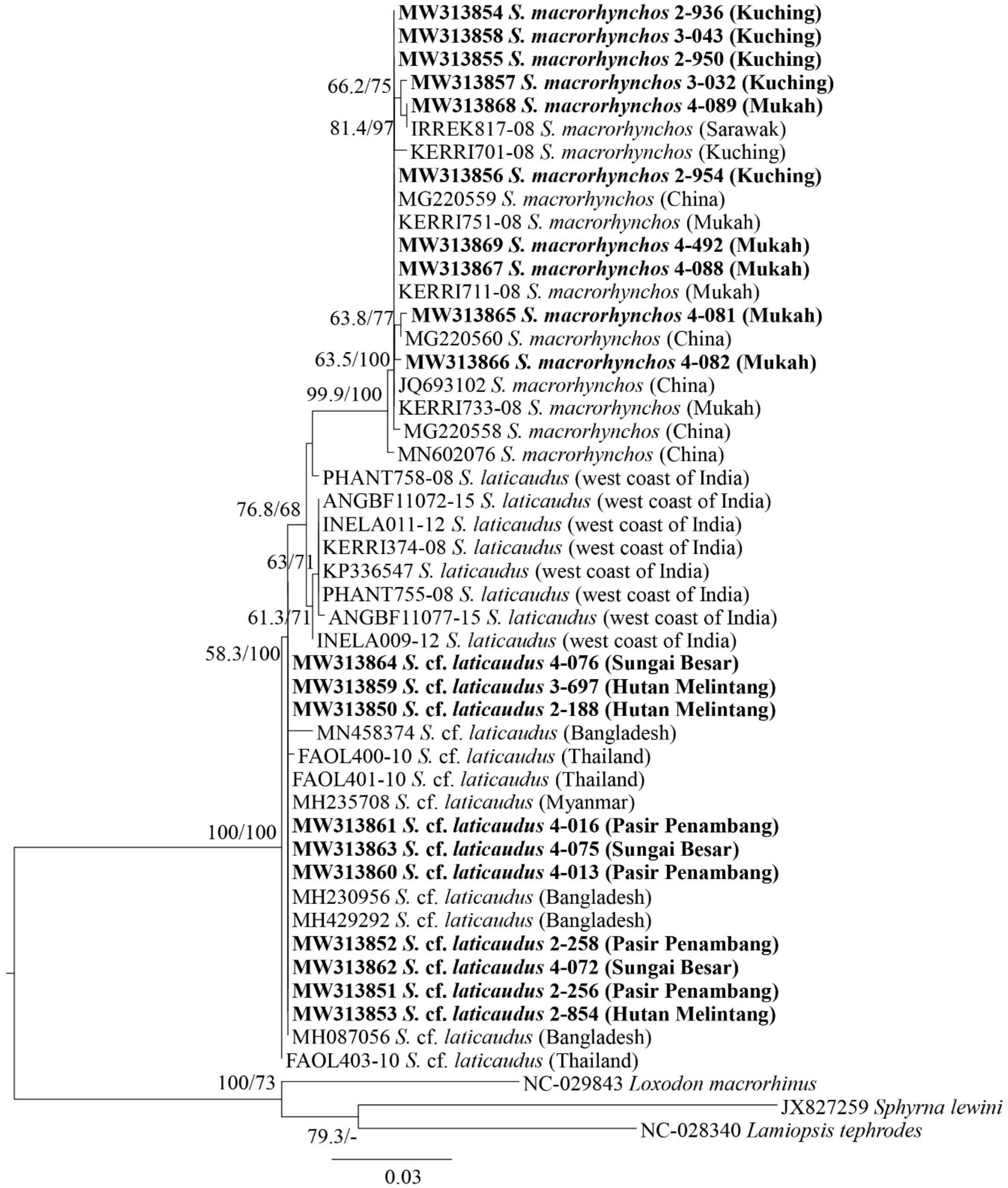

Figure S1a. *COI* gene mid-point rooting phylogenetic relationships of *Scoliodon* species (phylogram). The bootstrap values (ML/BI) are shown at branches. Sequence names in bold were from present study.

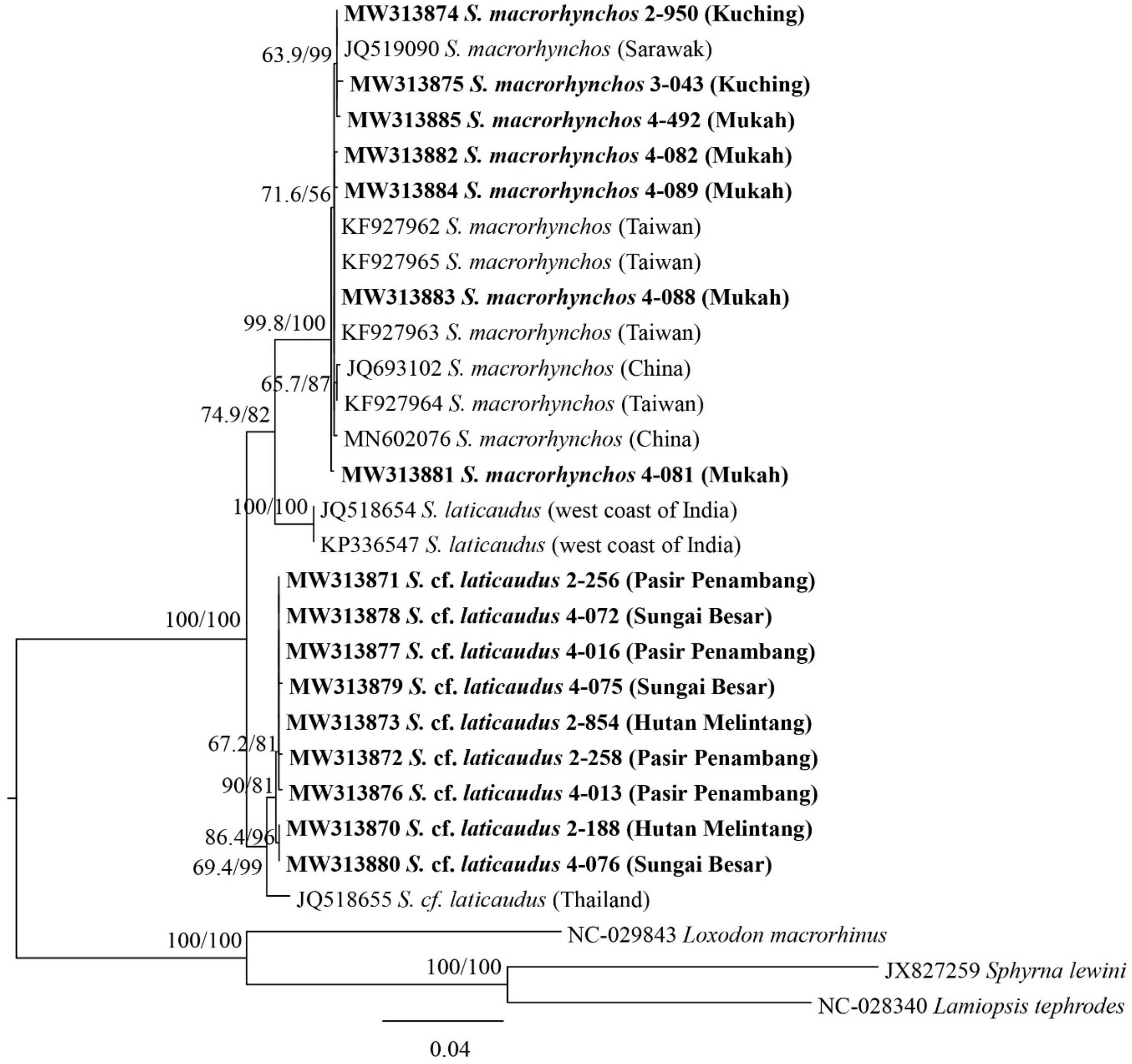

Figure S1b. NADH2 gene mid-point rooting phylogenetic relationships of *Scoliodon* species (phylogram). The bootstrap values (ML/BI) are shown at branches. Sequence names in bold were from present study.

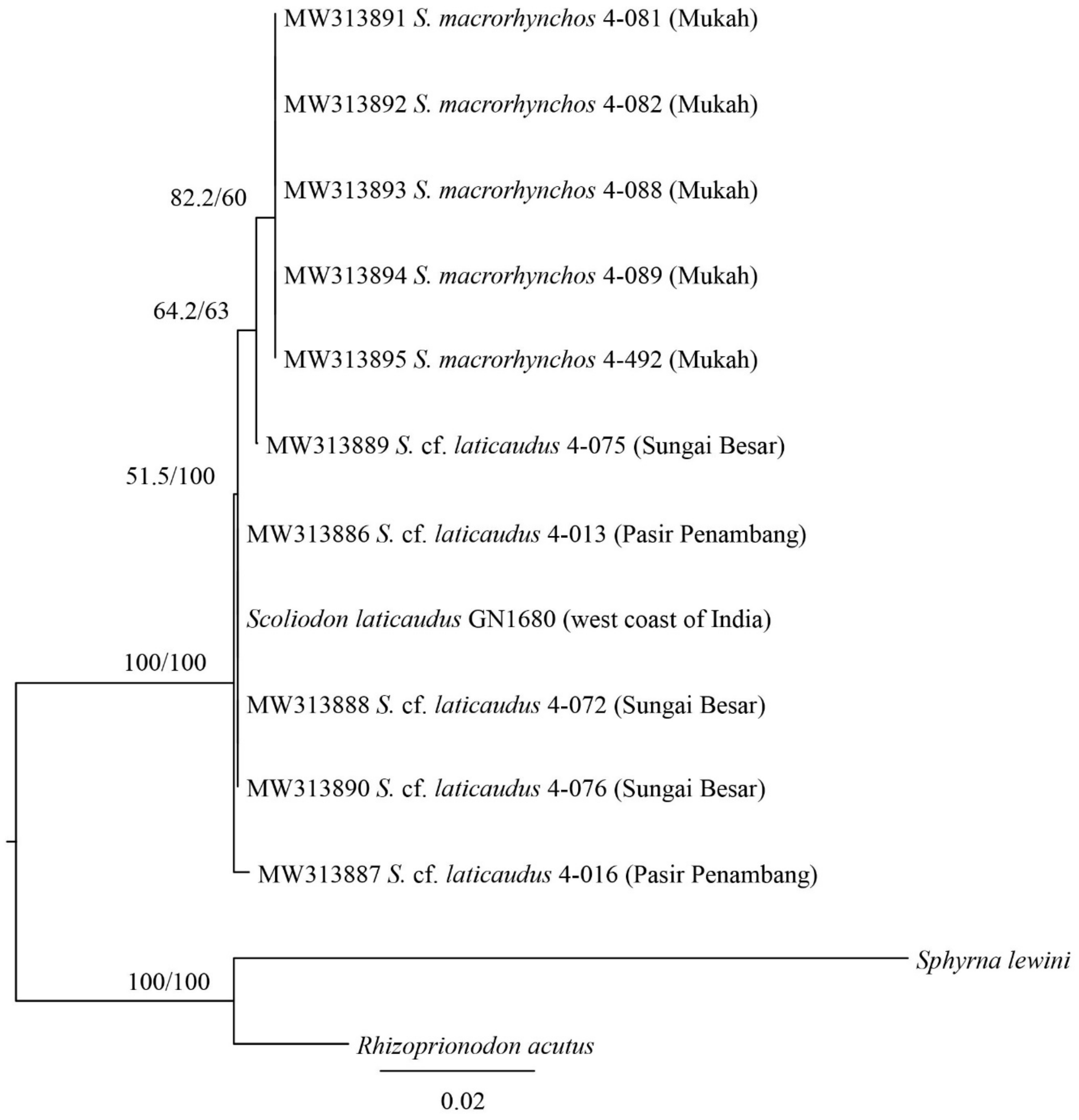

Figure S1c. ACT mid-point rooting phylogenetic relationships of *Scoliodon* species (phylogram). The bootstrap values (ML/BI) are shown at branches.

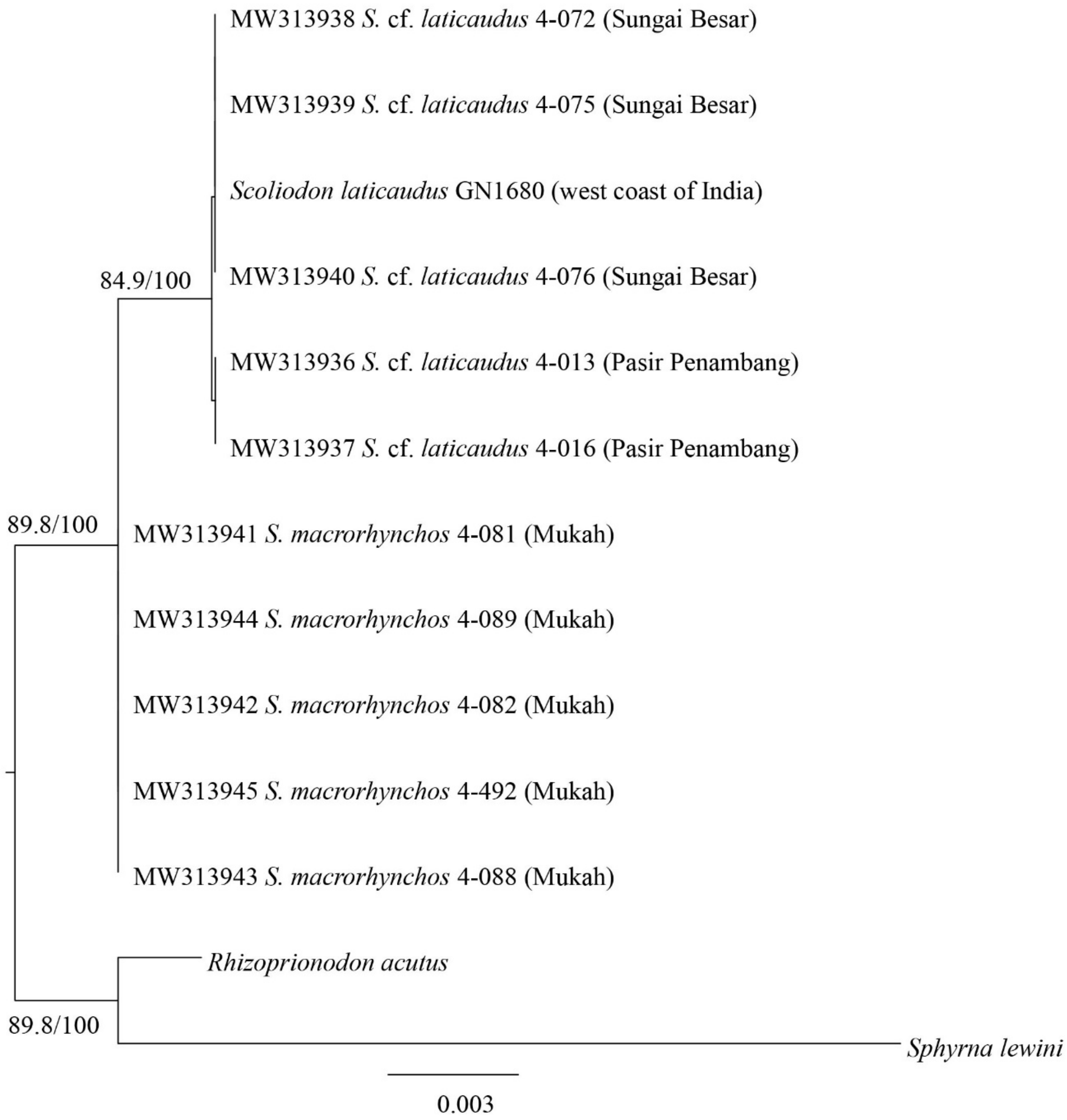

Figure S1d. KBTBD2 mid-point rooting phylogenetic relationships of *Scoliodon* species (phylogram). The bootstrap values (ML/BI) are shown at branches.

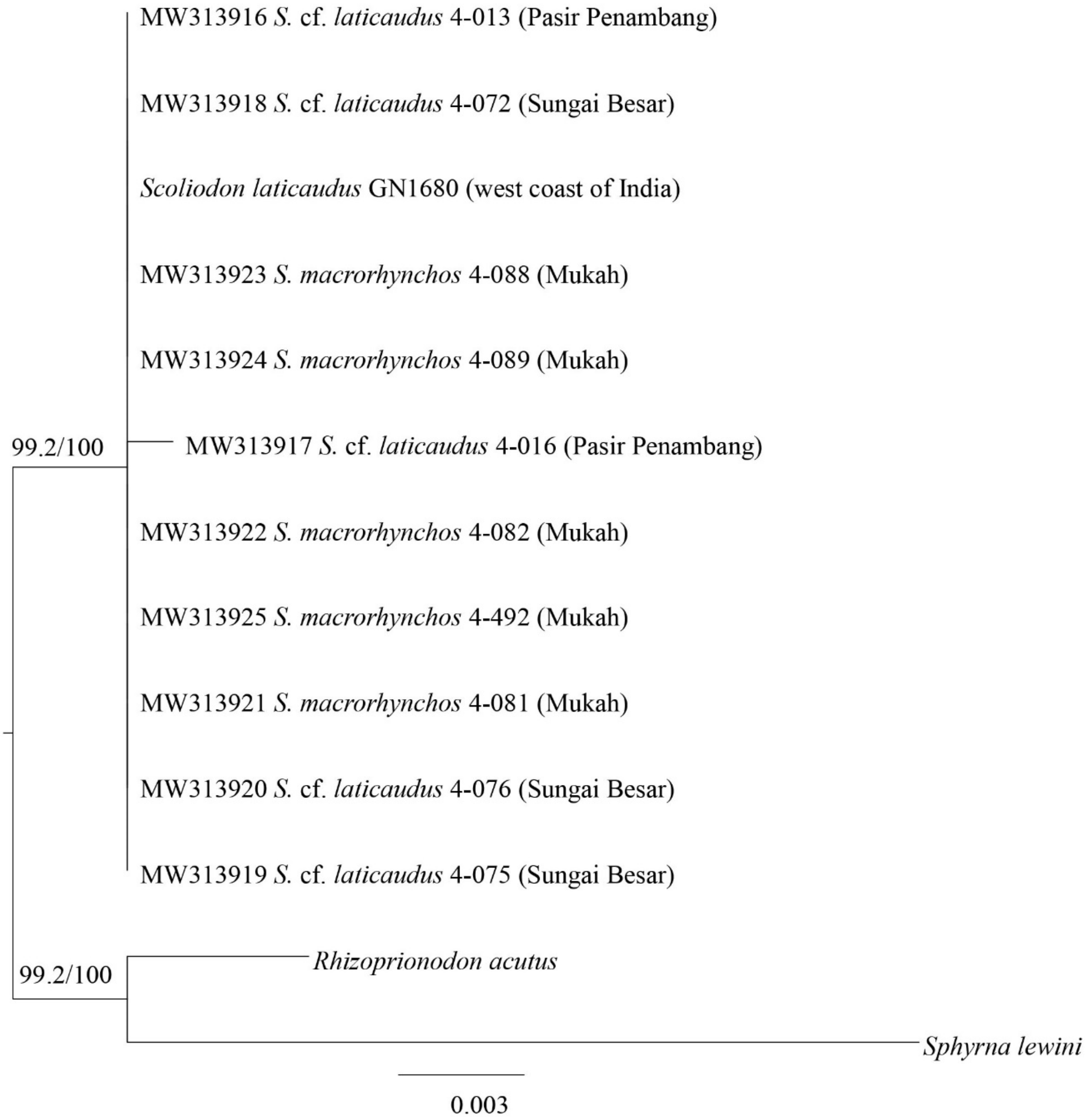

Figure S1e. PROX1 mid-point rooting phylogenetic relationships of *Scoliodon* species (phylogram). The bootstrap values (ML/BI) are shown at branches.

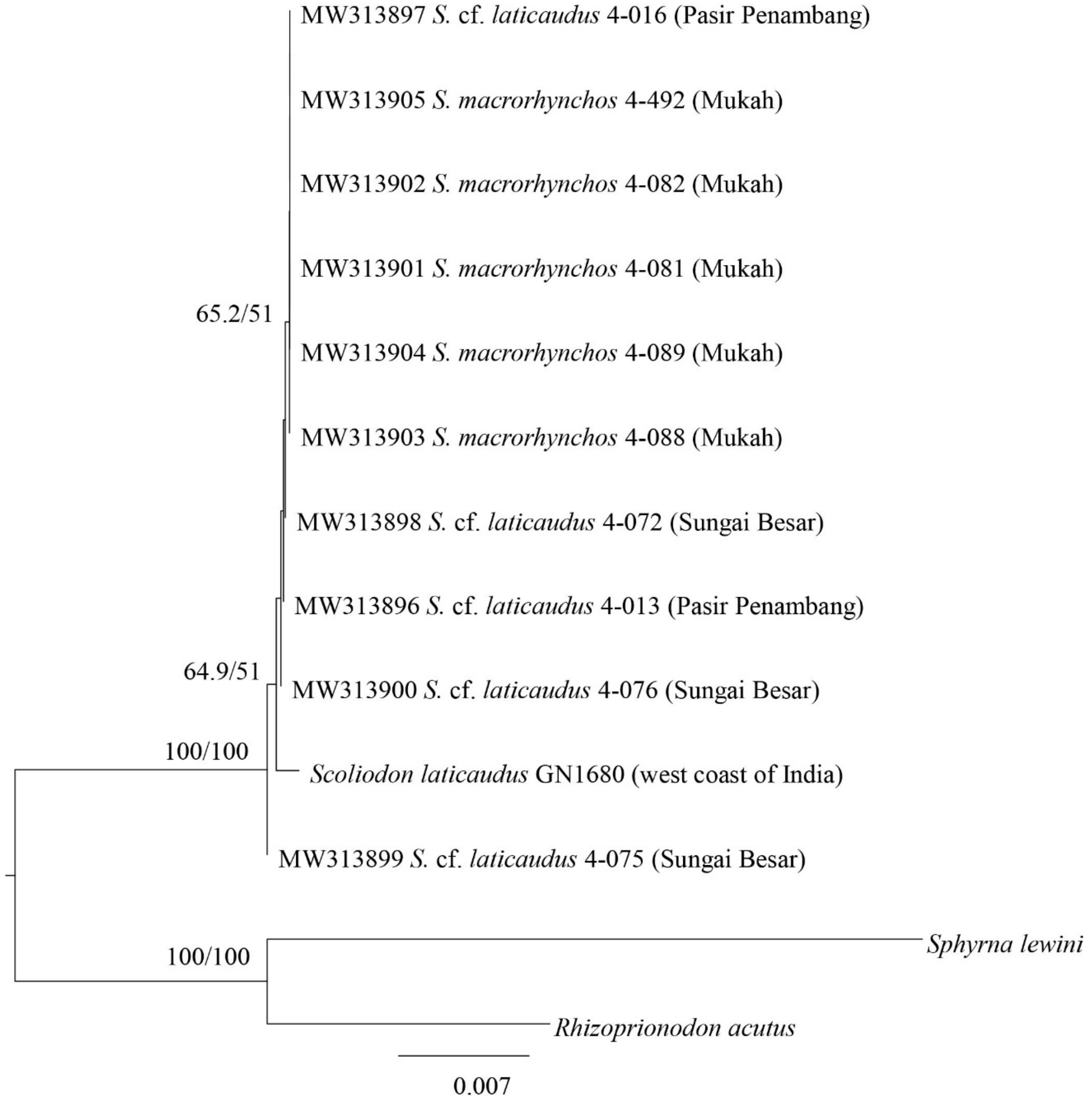

Figure S1f. RAG1 mid-point rooting phylogenetic relationships of *Scoliodon* species (phylogram). The bootstrap values (ML/BI) are shown at branches.

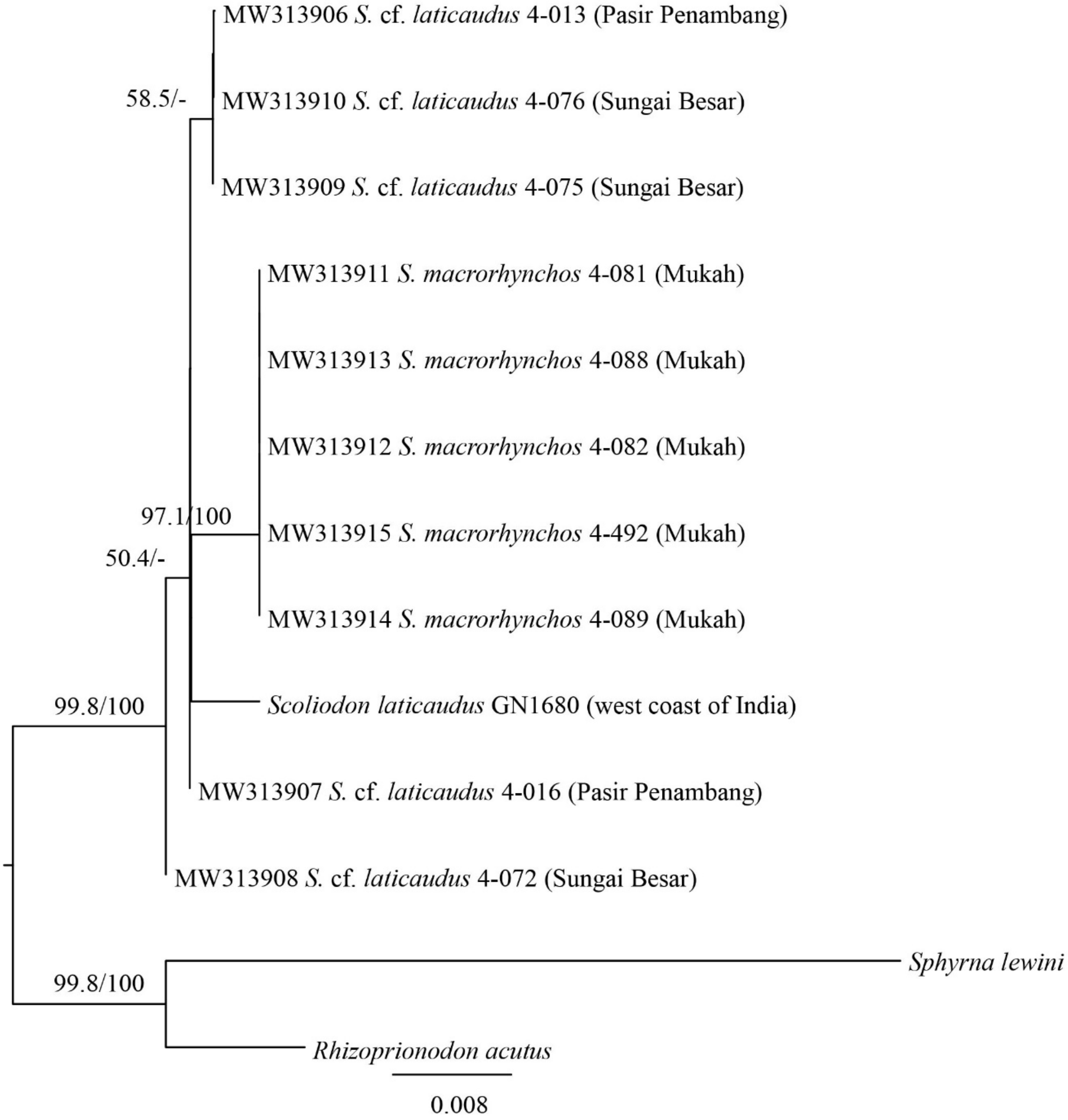

Figure S1g. RAG2 mid-point rooting phylogenetic relationships of *Scoliodon* species (phylogram). The bootstrap values (ML/BI) are shown at branches.

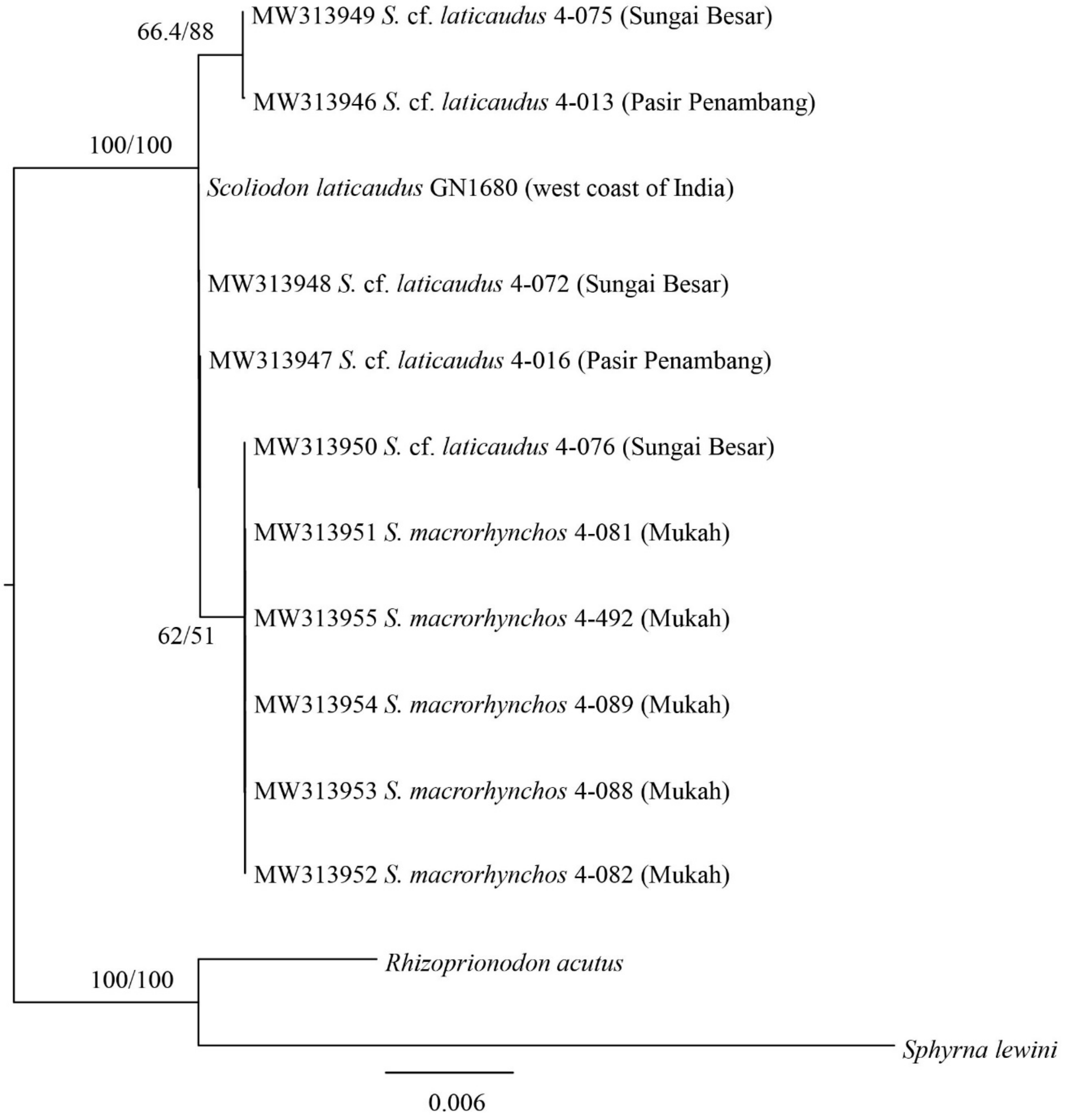

Figure S1h. SCFD2 mid-point rooting phylogenetic relationships of *Scoliodon* species (phylogram). The bootstrap values (ML/BI) are shown at branches.

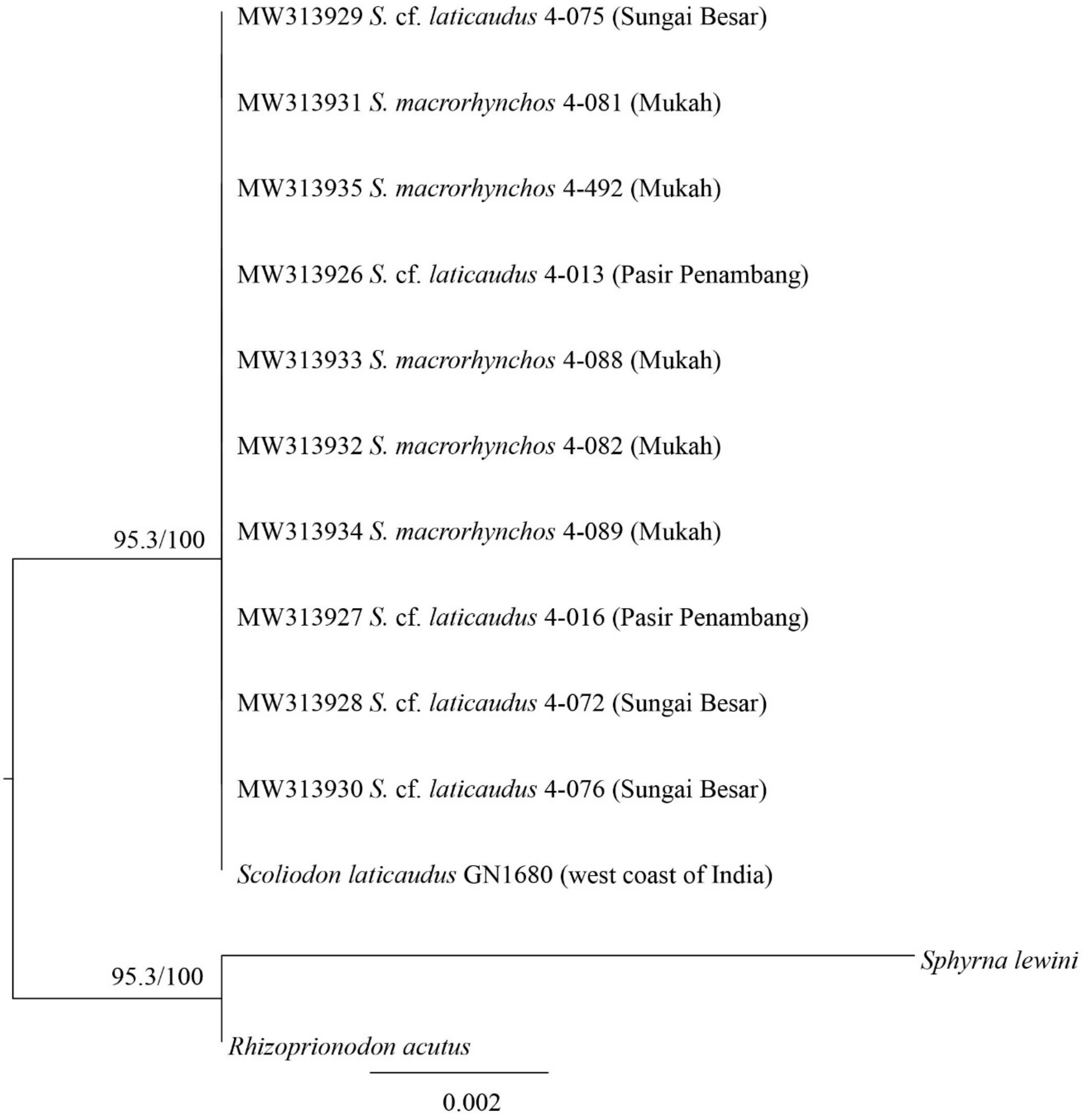

Figure S1i. TOB1 mid-point rooting phylogenetic relationships of *Scoliodon* species (phylogram). The bootstrap values (ML/BI) are shown at branches.
